# Quantifying hygroscopic deformation in lignocellulosic tissues: a digital volume correlation tool comparison

**DOI:** 10.3389/fpls.2025.1572745

**Published:** 2025-08-18

**Authors:** Kim Ulrich, Fabian Scheckenbach, Tak Ming Wong, Tom Masselter, Silja Flenner, Anaclara Visconti, Martin Nopens, Andreas Krause, Sergej Kaschuro, Jakob Benedikt Mietner, Thomas Speck, Imke Greving, Berit Zeller-Plumhoff, Linnea Hesse

**Affiliations:** 1 Plant Biomechanics Group @ Botanic Garden, University of Freiburg, Freiburg im Breisgau, Germany; 2 Cluster of Excellence livMatS @ FIT—Freiburg Center for Interactive Materials and Bioinspired Technologies, University of Freiburg, Freiburg im Breisgau, Germany; 3 Biomimetics Group, Institute of Wood Sciences, University of Hamburg, Hamburg, Germany; 4 Institute of Metallic Biomaterials, Helmholtz-Zentrum Hereon, Geesthacht, Germany; 5 Institute of Materials Physics, Helmholtz-Zentrum Hereon, Geesthacht, Germany; 6 Research Area Landscape Functioning, Leibniz Center for Agricultural Landscape Research (ZALF) e.V., Müncheberg, Germany; 7 Thünen Institute of Wood Research, Thünen Institute, Hamburg, Germany; 8 Data-driven Analysis and Design of Materials, Faculty of Mechanical Engineering and Marine Technologies, University of Rostock, Rostock, Germany

**Keywords:** biomechanics, wood science, lignocellulose, DVC, hygroscopy, computed tomography

## Abstract

Digital Volume Correlation (DVC) was used to study the hygroscopic shrinkage in lignocellulosic tissues. For this, small tissue segments of only a few cells were prepared from the endocarp of *Hura crepitans* fruits, the sclereid cell layer of *Pinus jeffreyi* pine cone scales, the sclerenchyma fiber sheath of peripheral vascular bundles in *Marantochloa leucantha* and latewood of *Pinus sylvestris*. The cells were imaged in a wet and dry state using X-ray nano-holotomography. Subsequently, a DVC analysis was conducted using Avizo™, elastix and MBS-3D-OptFlow, to visualize and quantify their hygroscopic shrinkage and to compare the accuracy of the approaches. The results reveal an anisotropic shrinkage behavior (1) along the cell length compared to radial shrinkage and (2) a greater radial than tangential shrinkage within the cell wall. The accuracy of the DVC results was validated and compared using two artificially deformed datasets (linear and sinusoidal) for controls. A (sub-)voxel accuracy for both controls could be demonstrated for each software with the image registration toolkit elastix performing best. In addition, the abundance of structural features in the cell walls leads to an improved DVC accuracy. Overall, DVC proved to be a viable approach to study the hygroscopic deformation of lignocellulosic tissue samples.

## 1 Introduction

The hygroscopic behavior of plant tissues is based on the ability to ab- and desorb water in response to changes in the ambient humidity. This process of ab- or desorption leads to swelling or shrinkage, respectively, resulting in deformation of the tissues. Differing rates of water ab- and desorption between different tissues can form stress gradients which may cause tissue ruptures or result in motion of plant organs. In some species, these behaviors are pivotal in facilitating seed dispersal, as is the case in the sandbox tree (Swaine and Beer, 1977), pine cones (Dawson et al., 1997) and the witch hazel (Poppinga et al., 2019). Such plant motions are subject of research for novel bio-inspired products like passive self-actuated façade shading systems (Wood et al., 2018; Li et al., 2021; Cheng et al., 2024). However, stress formation during hygroscopic deformation is also a challenge when considering technical applications based on plant materials (Glass and Zelinka, 2021). A targeted analysis of the hygroscopic deformation of different tissues is therefore of great interest, not only from a basic research perspective, but can also contribute to the improvement of bio-based and bio-inspired applications.

Therefore, the aim of this study is to present and evaluate novel approaches for quantifying water-induced deformations of different lignified plant tissues using synchrotron radiation-based nano-holotomography combined with Digital Volume Correlation (DVC). DVC is a technique to visualize and analyze the deformation of a sample as it transitions between two states. This approach will enable a deeper understanding of hygroscopic swelling and shrinkage on the cellular and tissue level and allows us to explore their potential implications for bio-inspired applications. For this we compare the DVC tools Avizo™ (commercial), elastix (open-source) and MBS-3D-OptFlow (open-source) based on four lignocellulosic tissues while evaluating their accuracy using artificial datasets as controls (Fischer et al., 2023).

In material science, a combination of computed tomography (CT) with DVC has been used for more than 20 years to capture internal structural changes and deformations during mechanical testing of materials (Bay et al., 1999; Fischer et al., 2023). However, this method is rarely used for biological samples, with the exception of biomedicine (Verhulp et al., 2004; Franck et al., 2011; Madi et al., 2020; Lavigne et al., 2022) and occasional wood research (Forsberg et al., 2008; Hardisty and Whyne, 2009; Hussein et al., 2012).

The hygroscopic deformation of wood microstructures is typically analyzed using conventional techniques and synchrotron radiation-based CT (SRCT) in combination with experimental setups for climate regulated scanning at adjustable temperature and relative humidity (RH) (Trtik et al., 2007; Derome et al., 2011; Rafsanjani et al., 2014; Patera et al., 2017, Patera et al., 2018; Florisson et al., 2023; Nopens et al., 2025). These scans can be analyzed with affine and non-affine image registration or digital volume correlation (DVC) techniques to quantify the sample deformation, but both of these techniques have limitations. In the context of image registration, the affine transformation accounts for global translation, rotation, shear, and scaling. In contrast, a non-affine transformation can allow for more complex distortions, such as non-linear scaling and warping. While affine image registration of wet and dry state CT images can be used to sufficiently analyze the global swelling and shrinking strain, it cannot resolve local deformations (Derome et al., 2011). Therefore, another approach is used to describe local deformations by employing non-affine 2D image registration, which aligns each slice of the reconstructed tomography data with its deformed counterpart, while assuming that longitudinal local deformations can be neglected and are adequately approximated by a global affine transformation (Patera et al., 2017, Patera et al., 2018). However, the anisotropy of some biological samples result in longitudinal local deformation which is of particular interest, e.g. for the anisotropic longitudinal elongation of pine cone scale tissues resulting in a bending movement (Dawson et al., 1997; Ulrich et al., 2024), the strain accumulation leading to the explosive seed dispersal of *Hura crepitans* L., Euphorbiaceae, (Gilles, 1905; Swaine and Beer, 1977; Ribera et al., 2020) or the behavior of reaction wood (Burgert et al., 2007; Patera et al., 2018; Eder et al., 2021).

On a subcellular level, hygroscopic swelling and shrinkage have been studied by analyzing cell-wall micropillars using SRCT and a combination of affine and non-affine 3D image registration (Rafsanjani et al., 2014). On a cellular level, however, it remains questionable if DVC algorithms can adequately describe the global and local swelling and shrinkage of lignocellulosic tissues with non-neglectable deformation along the z-axis. Thus, we analyzed different samples ranging from multiple cells to a single cut cell wall, with comparable sample dimensions using an identical experimental setup. In addition, we chose tissues with different functional purposes such as endocarp cells of an explosive fruit of the sandbox tree (*H. crepitans*), sclereid cells of a pine cone scale (*Pinus jeffreyi* Balf., Pinaceae), cells of a sclerenchyma fiber sheath of a monocotyledon (*Marantochloa leucantha* K. Schum., Marantaceae) and cells of pine latewood (*Pinus sylvestris* L., Pinaceae).

Today, a variety of algorithms and software enable DVC analysis (Powierza et al., 2019). The most common DVC approach is the so-called local approach that subsets the initial state (reference image) into subvolumes. The subvolumes of this reference image are then registered to the deformed state (deformed image) independently through a combination of the affine transformations: translation, rotation, shearing, and scaling. The fit is then evaluated by calculating a correlation metric based on voxel information and intensity distribution. Furthermore, a global approach DVC (GA-DVC) can be applied to analyze continuous deformations. In GA-DVC the deformation field is estimated using a finite-element (FE) mesh, akin to FE-methods. By pairing optical flow conservation with regularization techniques to maintain the mechanical elasticity, complex local and global deformations can be analyzed (Roux et al., 2008). The DVC extension XDigitalVolumeCorrelation in the commercial Avizo™ 3D (Thermo Fisher Scientific, Waltham, Massachusetts, United States) provides both a local and global approach (Powierza et al., 2019).

The open-source algorithm MBS-3D-OptFlow can be categorized as a variational method that solves the dense 3D optical flow problem based on the combined local-global approach. The MBS-3D-OptFlow algorithm was specifically developed to handle large image data from SRCT imaging (Schmelzle et al., 2021; Bruns et al., 2023). It has shown to be robust and has been optimized for GPU-acceleration. This algorithm was designed to allow abrupt changes, such as crack formation, in the deformation field by its anisotropic flow-driven regularizer and the volumetric image pyramid (Bruns et al., 2023). In order to deal with samples with low texture that are deformed beyond the image borders, options to set boundary conditions, i.e. pre-strains, have been implemented in the algorithm (Jungbluth et al., 2023).

DVC analysis similar to the global DVC approach can also be done using free-form 3D image registration. Image registration involves mapping a fixed (reference) image onto a moving (deformed) image based on rigid, affine and non-affine transformations, such as B-spline based transformations (Metz et al., 2011). As a result, a set of transformation parameters is acquired by iteratively minimizing a cost function based on a correlation metric via adjusting the transformation parameters. Based on these transformation parameters a voxel-based displacement field can be calculated. The open-source image registration toolkit elastix (Klein et al., 2010) combines such affine and B-spline transformations to perform DVC analysis of imaging data.

The aim of the presented study is to robustly quantify and visualize the anisotropic hygroscopic deformation of lignocellulosic tissues. We hereby compare three DVC approaches using Avizo™, elastix, and MBS 3D-OptFlow to evaluate their applicability, while estimating their accuracy using artificially deformed datasets.

## 2 Materials and methods

### 2.1 Samples

**Figure 1:**
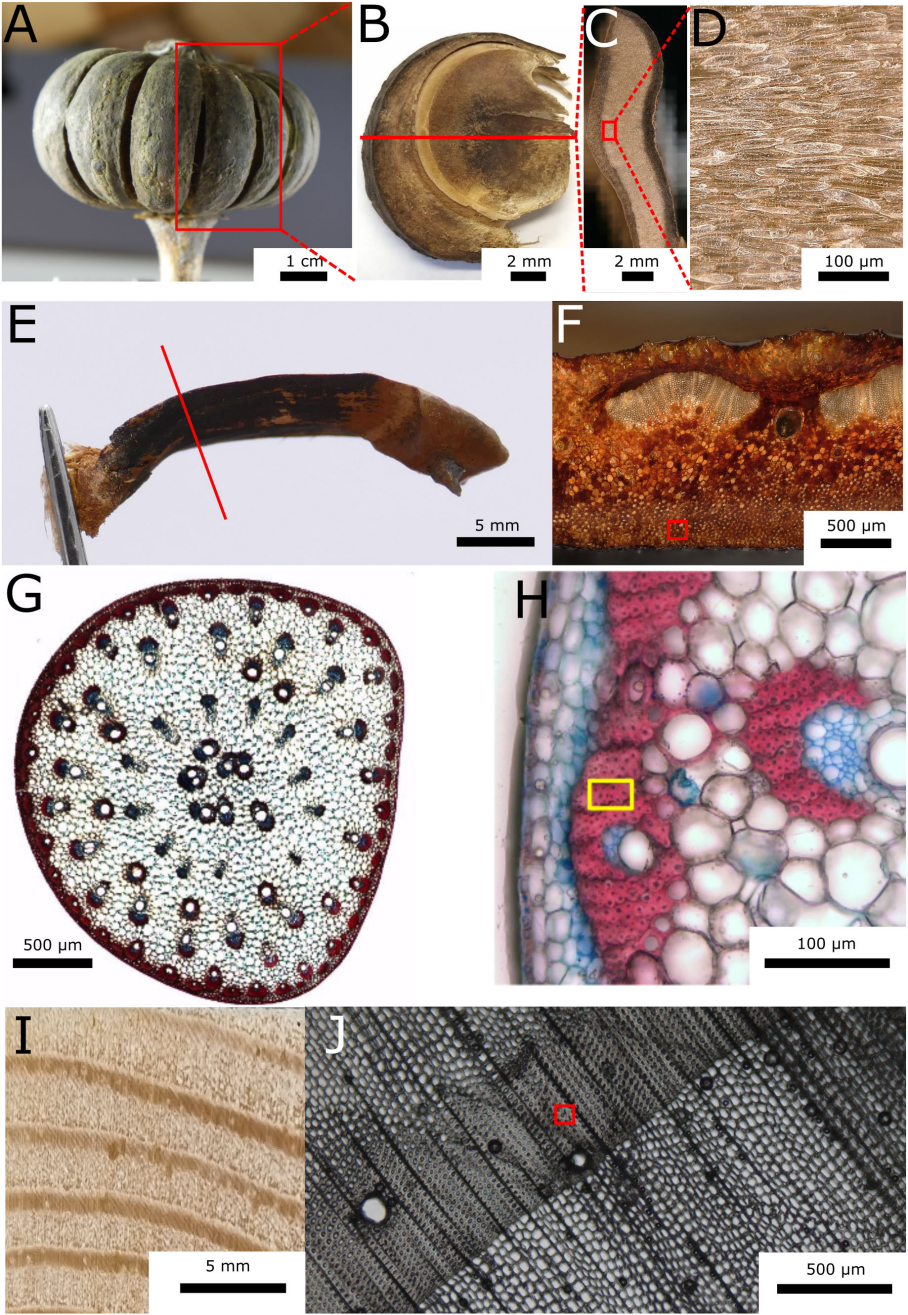
Tissue samples ranging in size from 10 to 40 µm were extracted from lignocellulosic tissues of four species. **(A)** *Hura crepitans* fruit. **(B)** Lateral view of a fruit carpel. **(C)** Cross-section of a fruit carpel. **(D)** Closeup of the endocarp tissue. **(E)** Side view of a *Pinus jeffreyi* pine cone scale. The red line indicates the position of the cross-sectional view in **(F)**. The sampling position is indicated by the red square in **(F)**. **(G)** Cross-section of a basal stem section below the first branching of *Marantochloa leucantha* stained using astra blue (non-lignified tissues are stained blue) and safranin (lignified tissues are stained red). **(H)** The yellow square indicates the sampling location in the sclerenchyma fiber sheath of a peripheral vascular bundle. **(I)** Photography of *Pinus sylvestris* wood (transversal section). **(J)** Light microscopic image of the cross-section of the wood. The sample is extracted from the latewood, as indicated by the red square.

One fruit (**Figure 1A**) from the sandbox tree (*H. crepitans*) was collected from trees grown in the greenhouses of the Botanical Garden in München-Nymphenburg in 2022. Individual carpels (**Figure 1B**) were transported to Hamburg where they were stored at room temperature. The sample used for this experiment was taken from the loculicidal, interior layer of a two-layered endocarp of one valve of a carpel (**Figures 1C, D**).

One pine cone of the yellow pine (*P. jeffreyi*) was collected in February 2022 from the Botanical Garden Freiburg after it had opened, dispersed most of its seeds and fell to the ground. The cone was then stored in a local laboratory at room temperature. Before further preparation, a single scale was separated from the cone and halved in the longitudinal direction. The sample used for the experiments was taken from the sclereid cell layer of the basal half of this scale (**Figures 1E, F**).

A basal stem section below the first branching of *M. leucantha* was collected in January 2023 in the Botanical Garden Freiburg. The cut stem section was stored in Eppendorf tubes in 70% ethanol. For the sample, sclerenchyma fibers of the bundle sheath of peripheral vascular bundles were isolated (**Figures 1G, H**).

A latewood sample (**Figures 1I, J**) was taken from a local scots pine tree (*P. sylvestris*) in Hamburg-Bergedorf that had been felled in February 2021. The cut cross-section of pine wood was stored in water in Hamburg at the Thünen Institute for Wood Research until sample preparation.

**Figure 2:**
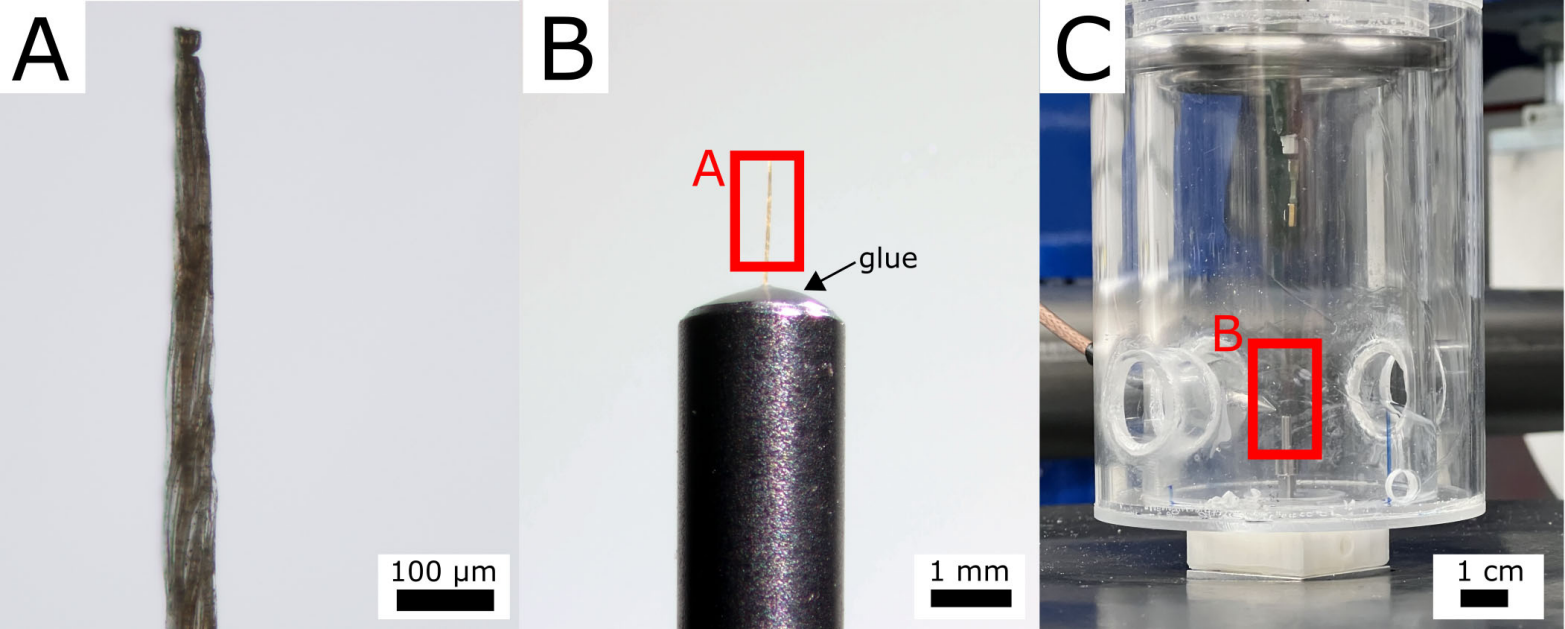
Scanning setup on the example of the *Hura crepitans* sample. **(A)** Light microscopy image of the sample. **(B)** Binocular image of the sample glued onto a sample holder using a UV repair system on the basis of plastic. **(C)** Experimental setup with the sample positioned in the climate chamber (Nopens et al., 2025).

All tissue samples were prepared within a maximum of two weeks prior to the imaging process. The cutability of the samples was improved by soaking them in water overnight. While still being soaked, they were cut into pillars measuring approximately 800 µm in length and 10-50 µm in width, following the longitudinal direction of the cells (**Figure 2A**). This preparation was conducted manually using a microscope (Olympus BX51, Evident Europe GmbH, Hamburg, Germany) and a razor blade. Subsequently, one end of the sample pillars was fixed to special sample holders (**Figure 2B**) using a UV-cured liquid plastic welding system (Bondic BC4000, VIKO UG, Kranzberg, Germany). The samples were then stored in a small container at room temperature until imaging.

### 2.2 Synchrotron radiation-based nano-holography with climate chamber

All samples were scanned using the nano-tomography system at the imaging beamline P05 operated by Helmholtz-Zentrum Hereon at PETRA III (DESY Deutsches Elektronen Synchrotron, Hamburg, Germany). The system offers transmission X-ray microscopy and near-field holotomography with high resolution down to below 50 nm. The setup is optimized for *in situ* applications, utilizing optics with relatively long focal lengths (Fresnel Zone Plates). It offers an adjustable energy range between 8 and 17 keV using a Si111 channel-cut monochromator.

The large sample-to-detector distance (20 m) allows for high magnification in the X-ray regime without requiring light-optical magnification. Therefore, a detector (Hamamatsu) can be used where the scintillator (10 µm GadOx) is directly mounted on the chip.

Image acquisition was performed using near-field holotomography, a phase contrast imaging technique. This configuration enables imaging of millimeter-sized samples across multiple length scales. In this study, a 300 µm gold Fresnel Zone Plate was utilized for focusing at an energy of 11 keV (Flenner et al., 2020b).

To control the relative humidity and temperature during image acquisition, a climatic chamber was specifically designed for the nano-tomography setup at P05 (**Figure 2C**; Nopens et al., 2025). The sample was positioned within the climate chamber, which was designed to enable unhindered nanotomography, thus enabling tomographic scans to be completed in mere minutes (Flenner et al., 2020a, Flenner et al., 2020b). The samples all varied in initial conditions due to sample history. Since drying close to 0% relative humidity can induce irreversible changes in the chemistry of the cell wall material, all samples were imaged first in the moist and then in the dry state.

Prior to imaging, the samples underwent conditioning at the respective relative humidity levels for 30 minutes to ensure equilibrium. The detector live view revealed an almost immediate hygroscopic response as the climate chamber settings were adjusted. After two to three minutes no further movement could be observed. All samples were scanned at 90% relative humidity (RH) followed by a second scan below 3% RH. Both scans were performed at an ambient temperature of 22°C. Before tomographic reconstruction, phase retrieval was performed on the image data using the artifact-suppressing reconstruction method (ASRM) implemented in the Holowizard framework (Dora et al., 2024a, Dora et al., 2024b). Tomographic reconstructions were then carried out using the GridRec algorithm (Dowd et al., 1999) with a Shepp-Logan filter, implemented in TomoPy (Gürsoy et al., 2014). A binning factor of 2 was applied to the *H. crepitans* sample prior to phase retrieval. The resulting voxel sizes were 116 nm for *H. crepitans* fruit, 61.6 nm for the *P. jeffreyi* pine cone scale and *M. leucantha* sclerenchyma fiber, and 100 nm for the *P. sylvestris* latewood sample.

### 2.3 Pre-processing

All reconstructed volumes were pre-processed using FIJI (Schindelin et al., 2012) following these steps: (1) Due to deformation and elongation of the samples along the z-axis between climate steps, it was necessary to define a common region of interest (ROI) along the z-axis for further analysis. The extent of overlap between the images was optically estimated by identifying unique landmarks present in both the wet and dry states of a sample and then the common ROI was extracted. (2) To minimize image size and reduce computation time, the volume in the x-y plane was cropped to remove surrounding air, and the pixel data type was converted to 8-bit. (3) The images were then masked by first creating a binary mask of the remaining air based on the Intermodes method, and then (4) by subtracting the mask from the original image using the image calculator. (5) A rigid image registration was performed using elastix (Ver. 5.1.0) to coarsely align the wet and dry state images. (6) The resulting, aligned and masked volumes were then saved and imported into the DVC software. A step-by-step instruction to an alternative pre-processing using Avizo™ can be found in the **Supplementary Data**.

### 2.4 Local and global digital volume correlation – Avizo™

DVC in Avizo™ (Version 2024.1) offers a method for combining affine registration with tetrahedral mesh capabilities to calculate a continuous displacement field. The DVC involves two approaches: A local (LA-DVC) and a global approach (GA-DVC). In this study, we combined the LA-DVC and GA-DVC as suggested in the manual, using the displacement field generated by LA-DVC as an initial guess for the GA-DVC.

The LA-DVC module in Avizo™ is used to individually register sub-volumes of the pre-processed images to compute a coarse displacement field from the wet state (> 90% RH) to the dry state (< 3% RH) using rigid transformations. An edge length of 100 voxels (approx. 10 µm across all samples) was selected for all samples to calculate the displacement. Avizo™ utilizes a hierarchical approach for the computation, beginning with a coarse resampling of the reference volume, and proceeding to finer resolutions. Among the various measures of similarity, including Euclidean distance, mutual information, and normalized cross-correlation coefficient (NCC), the NCC was selected for this work.

Next a FE-based mesh was constructed enveloping the whole sample to compute the GA-DVC. Although the mesh can be adapted to conform to the sample structure, a dilated convex hull was employed in this study to maintain the continuity of the displacement field and ensure comparability between the different Digital Volume Correlation (DVC) approaches. A coarse tetrahedral mesh with an edge length of 100 voxels and a fine mesh with an edge length of 50 voxels were constructed using Avizo™. Due to the nature of tetrahedral grids, tetrahedral elements are smaller in volume compared to cubic grids (hexahedron) with the same edge length. Thus, the estimated accuracy of a tetrahedral mesh with an edge length of 50 voxels is comparable to a cubic grid size of 25 voxels. Upon applying the GA-DVC module on the FE-meshes, the mesh nodes act as reference points for calculating the displacement field, following the principles of optical flow conservation (Horn and Schunck, 1981; Mitiche and Bouthemy, 1996). Optical flow conservation for 3D grey field images is maintained by minimizing the following, linearized Taylor expansion (Roux et al., 2008) (Equation 1):

(1) $$\tilde{T}\left(U\right)=\;{∭}_{D}{\left[ɡ\left(x\right)-f\left(x\right)-\nabla f\left(x\right)*U\left(x\right)\right]}^{2}dx$$

With g(x) being the deformed image, f(x) the reference image, ∇f(x) the partial derivatives of f and U(x) the displacement.

To enhance computational efficiency, a linearized function is used in Avizo™, despite the nonlinear nature of reality, which encompasses multiple secondary minima. These secondary minima are addressed by constraining the search for functions in the displacement field and rigorously filtering the texture of both the reference and deformed volumes (Roux et al., 2008). This Taylor expansion is adapted for first order shape functions, such as 4-node tetrahedral (T4), as is the case in Avizo™ (Madi et al., 2013). As outlined in the user manual, the initial displacement field for the GA-DVC is obtained from the LA-DVC (Avizo, 2021). However, since the LA-DVC does not resolve displacements at the same coordinates as the T4 mesh, Avizo™ applies trilinear interpolation to adjust the displacement field accordingly.

To execute the GA-DVCs on the previously generated meshes, the following parameters were chosen: 500 iterations, a convergence criterion of 0.001 and the standard regularization factor of twice the mean edge length of the mesh. In Avizo™, the mechanical regularization applies an elastic assumption upon calculating the displacement field, allowing for smaller grid sizes and less detailed microstructural information (Avizo, 2023). For large local deformations, a low regularization should be chosen, while for homogeneous deformations a larger regularization reduces the computation time drastically by smoothing out local minima. To further minimize errors caused by local minima, it was essential to perform an iterative approach from a coarse to a fine mesh. Since the resulting displacement vectors are based on the nodes of the T4 mesh and not dense, we interpolated those displacement vectors onto the voxels of the reference image using trilinear interpolation for further analysis. A detailed step-by-step instruction for the DVC approaches in Avizo™ can be found in the **Supplementary Data**.

### 2.5 Digital volume correlation with optical flow – MBS-3D-OptFlow

MBS-3D-OptFlow method can be formulated as solving the anisotropic flow-driven optical flow problem by minimizing the energy function E for the dense 3D deformation field u (Equation 2):

(2) $$\begin{array}{c} E\left(u\right)=E_{data}\left(u\right)+\alpha E_{smooth}\left(u\right) \end{array}$$

with α denoting the smoothness regularization coefficient. For more theoretical details, readers can refer to Schmelzle et al. (2021) and Bruns et al. (2023).

Regarding the experimental setup, the publicly available implementation from Github (https://github.com/brunsst/MBS-3D-OptFlow) is adopted based on our use cases. Parameters (i.e. smoothness, gaussian pyramid, intensity normalization, derivatives, filtering) are optimized by maximizing the cross-correlation score and minimizing the mean of square difference between the reference and the estimated deformed volume. The optimal parameters (except for intensity normalization) are empirically fine-tuned based on the endocarp of a *H. crepitans* fruit for linear and sinusoidal controls, and then applied to all 4 samples. For the intensity normalization, the measured datasets of 4 samples have a similar intensity level, while the linear control and sine control datasets have different intensity levels. Therefore, a simple linear intensity normalization (i.e. normalizing [0, 255] to the range [0, 1]) is applied to the measured datasets, and the independent histogram normalization (i.e. normalizing 99.9% dynamic range of the cumulative histogram to the range [0, 1] for the reference and deformed volumes independently) is applied to the linear and sinusoidal controls datasets.

As the inner structure of the samples does not contain rich texture information, optical flow algorithms can lead to zero-deformation in its data term. Hence, a gradient masking feature of MBS-3D-OptFlow is employed by defining the top 20% of gradient magnitude as the region-of-interest of data term. Moreover, as the deformation fields are expected to be smoother than the example shown in the repository, the weight of smoothness term is increased to 0.15. The volume pyramids are also enlarged to 49 levels. The pyramid scales are set for four samples respectively, i.e. scale = {0.9643, 0.9595, 0.9504} corresponding to the z-dimensions of data at {1024, 1300, 2048}, such that the top-level volume pyramid is down sampled to 178 voxels at its z-axis dimension. Only a Gaussian pre-filtering is applied to the volume using sigma = 0.5.

### 2.6 Digital volume correlation with image registration – elastix

The image registration software elastix (Version 5.1.0) uses features of the Insight Toolkit (ITK, Yoo et al., 2002; McCormick et al., 2014) and incorporates various non-rigid methods such as affine and B-spline transformations (Metz et al., 2011) to align 2D and 3D images. In our use case we utilized the built-in option to stepwise combine different transformations. Two images were first aligned using an affine transformation to address large global deformations, after which a B-spline transformation is applied to capture local deformations. The affine transformation was applied with a three-level smoothing image pyramid and 500 iterations per level. Afterwards, the B-spline transformation was performed with a final control grid spacing of 25 pixels and a smoothing image pyramid comprising five resolution levels, with 1,000 iterations per level. Both the affine and B-spline transformation were carried out utilizing a random image sampler with 100,000 sampling positions to calculate a NCC coefficient. The randomized image sampler in elastix reduces computation time while maintaining accuracy comparable to that of a full image sampler (Fischer et al., 2023).

During runtime, the algorithm solves an optimization problem by minimizing a cost function C, which is based on the two images $I_{M}$ and $I_{F}$, the selected correlation metric as a similarity measure for the two images and a transformation $T_{\mu }$, with the optimization being performed over the parameters μ of the transformation $T_{\mu }$ (Klein et al., 2007, Klein et al., 2010) (Equation 3):

(3) $$\begin{array}{c} \hat{\mu }=\text{a}\text{r}\text{g}\underset{\mu }{\min}C\left(\mu ;I_{F},I_{M}\right) \end{array}$$

Affine transformations describe the deformation of the sample from a global perspective (Klein et al., 2010) (Equation 4):

(4) $$\begin{array}{c} T_{\mu }\left(x\right)=Ax+t \end{array}$$

With x being the image pixels, t the translation vector and A being a matrix, that can translate, rotate, scale and shear the image. A B-spline transformation (Metz et al., 2011) is based on B-splines (Rueckert et al., 1999) with a grid of control points positioned on a regular grid across the fixed image. This transformation lies between global and local transformations (Loeckx et al., 2010), providing a flexible and smooth mapping between the images (Equation 5):

(5) $$\begin{array}{c} T_{\mu }\left(x\right)=x+\sum_{x_{k}\in {\text{N}}_{x}}p_{k}\beta^{3}\left(\frac{x-x_{k}}{\sigma }\right) \end{array}$$

With $x_{k}$ being the control points, $\beta^{3}\left(x\right)$ the cubic multidimensional B-spline polynomial (Unser, 1999), $p_{k}$ the B-spline coefficient vectors, σ the B-spline control point spacing and $N_{x}$ the set of all control points. By changing the transform parameters, the similarity of the reference and the transformed image is evaluated, and the corresponding cost function is minimized. Upon completion, elastix stores the transformation parameters in a separate file, which can subsequently be transferred to transformix for computing the dense displacement field image based on the transformation parameter file. A detailed step-by-step instruction for the DVC approach in elastix can be found in the **Supplementary Data**.

### 2.7 Strain calculation and visualization

The Python (Version 3.11.9) implementation of the Insight Toolkit strain filter extension (McCormick, 2017, ITK version 5.3.0, itk-strain version 0.4.0) was employed to calculate the Green-Lagrangian strain tensor E based on the resulting displacement fields (Equation 6):

(6) $$\begin{array}{c} \text{E}=\frac{1}{2}\left(F^{T}*F-I\right) \end{array}$$

**Figure 3:**
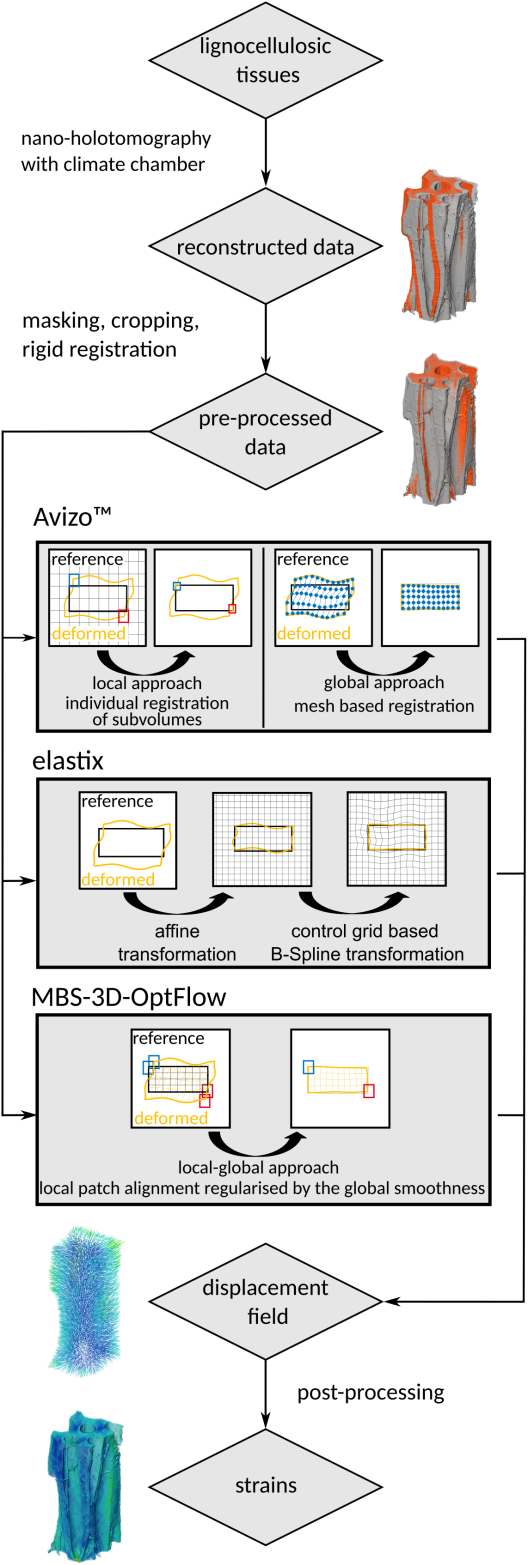
Visual representation of the workflow from lignocellulosic tissues to strain fields using Avizo™, elastix, and MBS-3D-OptFlow.

With F being the deformation gradient and I the identity matrix. For simplicity, only components $\epsilon_{xx}$, $\epsilon_{yy}$ and $\epsilon_{zz}$ of the strain tensors along the cell axis were used for analysis and visualization. The resulting strain fields display the estimated local strain from the wet state (RH > 90%) to dry state (RH "<" 3%) conditions. Since the masked images contain background in the cell lumina and the surrounding of the samples, the DVC also mapped these regions between the images. We applied a mask based on the bulk of the wet-state sample to filter the resulting strain fields for the relevant data. The results were plotted using matplotlib (Hunter, 2007, Version 3.8.4). A statistical comparison of the voxel-based axial strain results obtained from the three approaches was not performed due to the large sample size (voxels), which renders even negligible differences statistically significant. This is illustrated in **Supplementary Figure S1**. The complete workflow from lignocellulosic tissue to analyzed strain fields is illustrated in **Figure 3**.

### 2.8 Manufactured solution

Using the dry state images of our samples as a basis, we created two artificially deformed images to analyze the accuracy of the different DVC software. The artificially deformed images were generated using ITK. This process involved first generating a continuous displacement field using NumPy (Harris et al., 2020, Version 1.26.4), and then applying the corresponding deformation to the original images based on this field. The displacement vector components along the x-, y-, and z-axes of the applied displacement field were defined as follows (Equation 7):

(7) $$\begin{array}{c} {\tilde{u}}_{i}\left(x\right)=\sin(2\pi *\frac{c_{i}-x}{L})*A+\left(x-c_{i}\right)*E_{i} \end{array}$$

With i indicating the axial component, x the voxel position along the respective axis, $c_{i}$ the center along the respective axis, L and A the wavelength and amplitude of the sinusoid and E the axial gradient. For the first displacement field (linear control), A was set to 0 along all axes to compute a linearly expanded artificial image that is comparable to the wet-state image of our real image pair. For the second displacement field (sinusoidal control), A was set to 10 for the z component and 5 for the x and y components. $E_{z}$ was set to be -0.05 and $E_{x}$ and $E_{y}$ to -0.10 for both displacement fields. Due to the nature of image deformation with ITK, a negative gradient was applied to “swell” the dry-state images. To replicate the statistical noise between scans and the shifts in grey values caused by water absorption during the real measurements, Gaussian noise with a mean of 30 grey values and a standard deviation of 3 grey values was added to the artificially generated image. These values represent the upper limits of the observed grey value shifts in the actual image pairs, as estimated by manually checking the grey value distribution in the sample bulk.

These artificial image pairs were then analyzed with the same workflow as the real image pairs using Avizo™, elastix and MBS-3D-OptFlow. The Euclidean distance between the DVC-estimated and theoretical displacement field was calculated for each voxel of the reference volume and the resulting Euclidean distance field was used for further visualization.

## 3 Results

### 3.1 Synchrotron radiation-based nano-holotomography

**Figure 4:**
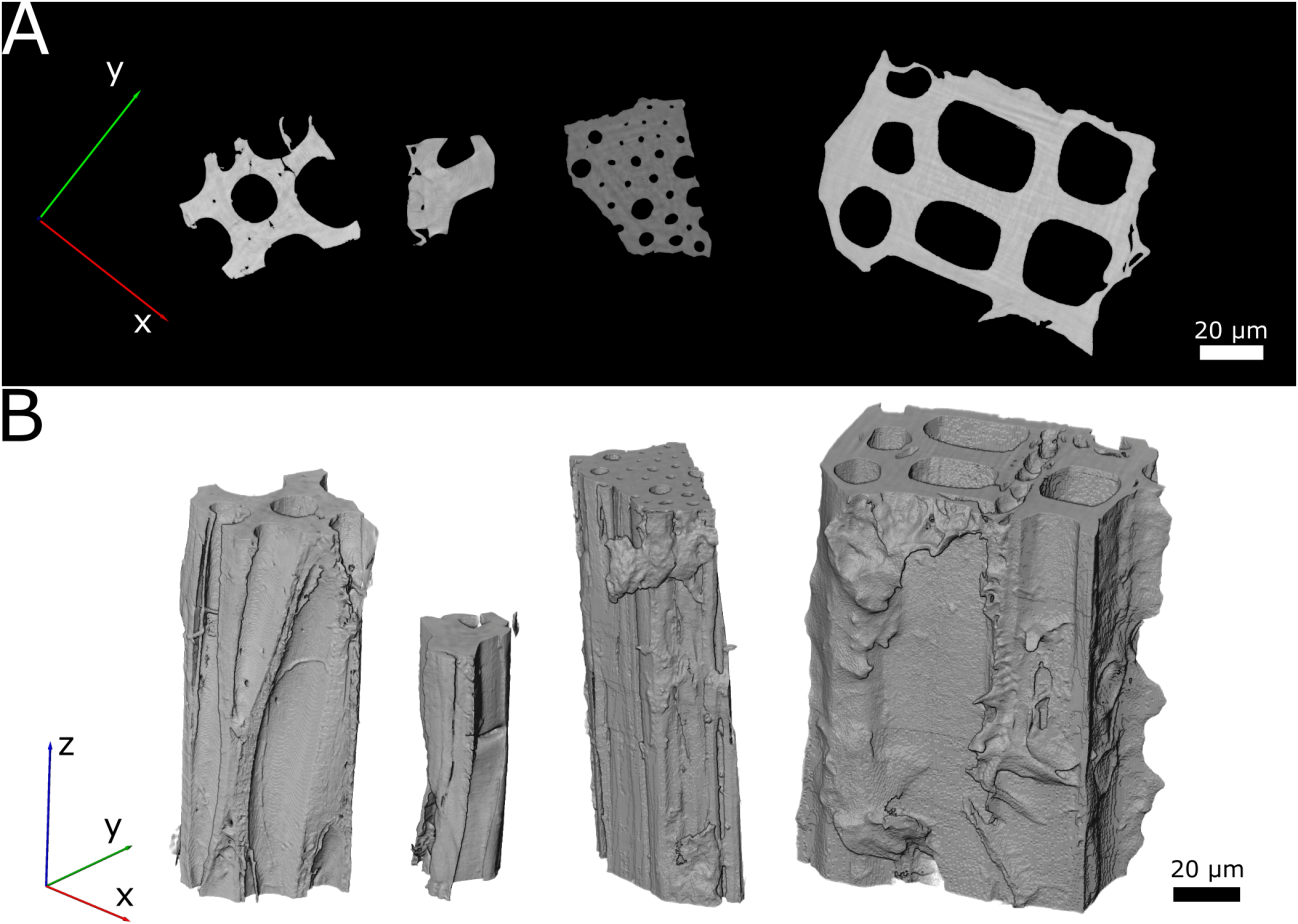
Visualization of the scanned wet state samples. From left to right: endocarp of *Hura crepitans* fruit, sclereid cell of *Pinus jeffreyi* pine cone scale, sclerenchyma fiber sheath of *Marantochloa leucantha* and latewood of *Pinus sylvestris*. **(A)** Cross-section image taken at 50% height of each sample. The specific images correspond to Slice 512 for the *H*. *crepitans* fruit, Slice 650 for the sclereid cell of *P. jeffreyi* pine cone scale, Slice 1024 for the sclerenchyma fiber sheath of *M. leucantha* and Slice 650 for the latewood of *P. sylvestris*. **(B)** Volume renderings of the scanned samples.

The four samples have different structural characteristics, as evidenced by the variation in cell size and number observed in the scans (**Figure 4**). The endocarp sample of the *H. crepitans* fruit consisted of an intact cell surrounded by several partially cut cell walls. The sclereid tissue sample of a *P. jeffreyi* pine cone scale comprised a single partially cut cell wall, exhibiting only a few landmarks (e.g., one pit vs many pits in the *H. crepitans* sample) and a minimal microstructure discernible in the reconstruction. The *M. leucantha* and *P. sylvestris* samples displayed multiple, densely packed cells. The abundance of pits and other landmarks were comparable to the pine cone sample.

### 3.2 DVC Analysis of hygroscopic material behavior

**Figure 5:**
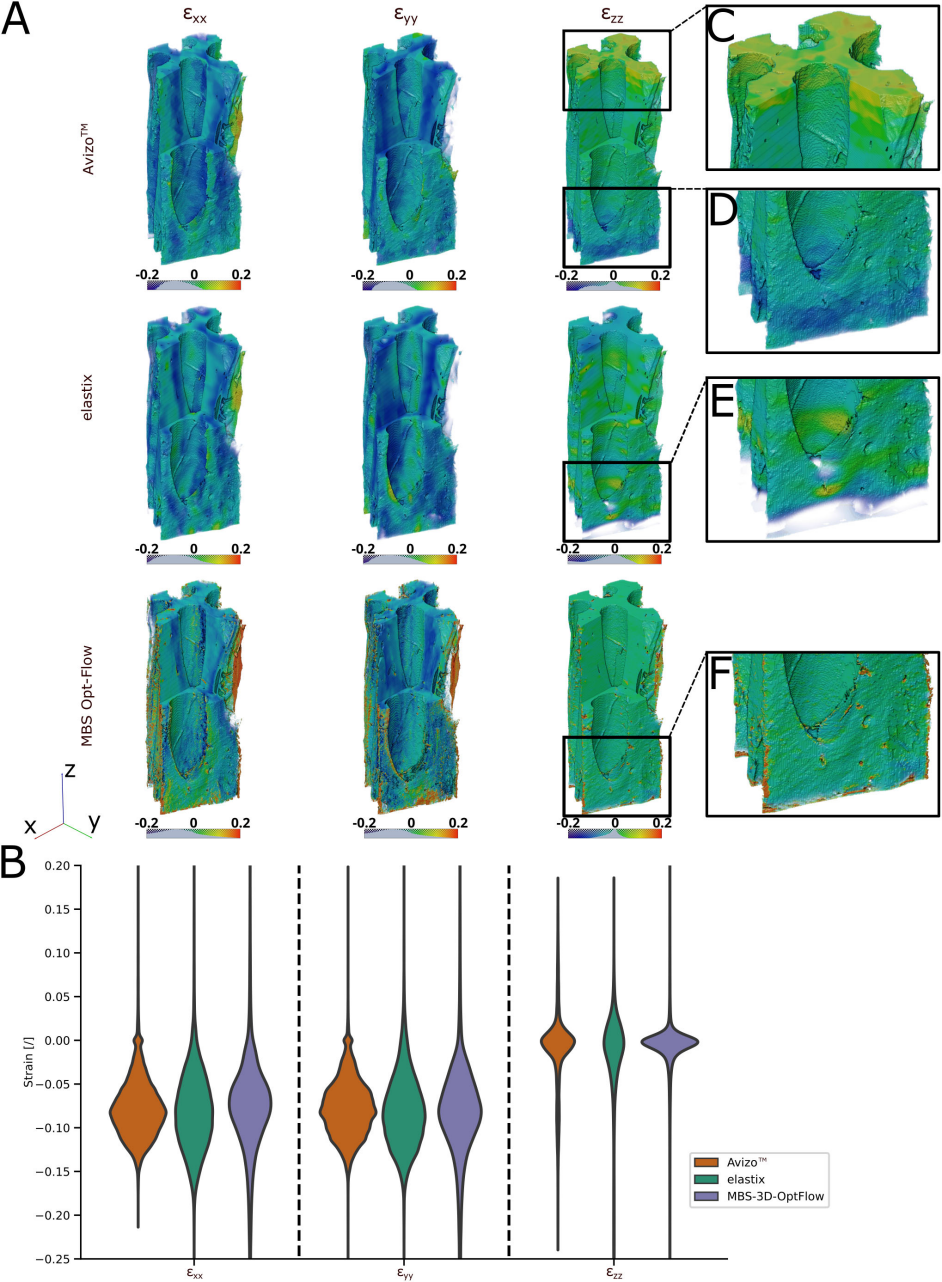
3D visualization and violin plot of the DVC analysis using Avizo™, elastix and MBS-3D-OptFlow. **(A)** Volume rendering of the masked x-, y-, and z-axial components of the Green-Lagrangian strain of the *Hura crepitans* sample with a visual cropping of the front corner to display interior strains. Each image row distinguishes one of the three software used. In case of Avizo™, the upper- and lowermost slices, which were not covered by the mesh, and thus contain strain values of zero are cut off. **(B)** Distribution of the axial strains across a randomized subsample of 100,000 voxels of the bulk of the reference image sample. **(C, D)** The results of Avizo™ and MBS-3D-OptFlow show higher and lower strains towards the extremities of the z-stack when compared to elastix. **(E, F)** The results of elastix and MBS-3D-OptFlow show strain peaks around pits in the cell wall.

In the following, we limit the results to a presentation of the *H. crepitans* fruit sample due to the considerable quantity of data. **Table 1** provides a summary of the results for the other samples and detailed visualizations are available in **Supplementary Figures S2**-**S4**. During the interpolation of the node-based displacement field and the calculation of the strain field for the Avizo™ results, errors occurred in the form of infinite values at the edges of the sample bulk. These values were filtered out prior to further analysis and visualization. As a result, the number of eligible voxels for the calculation of the mean strains was reduced for all Avizo™ results. The upper- and lowermost slices of the Avizo™ results contained only strain values of zeros as they were not covered by the mesh, which can be seen as a peak at 0% strain in **Figure 5B**.

**Table 1:** Median values (and inter quartile range) of axial strains calculated across the voxels of the masked sample bulk.

Sample	Avizo™			elastix			MBS-3D-Opt-Flow		
	ϵ_xx_	ϵ_yy_	ϵ_zz_	ϵ_xx_	ϵ_yy_	ϵ_zz_	ϵ_xx_	ϵ_yy_	ϵ_zz_
*H. crepitans* fruit	-0.081 (-0.101, -0.059)	-0.079 (-0.100, -0.057)	-0.004 (-0.017, 0.007)	-0.083 (-0.112, -0.053)	-0.084 (-0.111, -0.056)	-0.007 (-0.028, 0.011)	-0.075 (-0.104, -0.049)	-0.081 (-0.11, -0.053)	-0.003 (-0.01, 0.002)
*P. jeffreyi* pine cone scale sclereid cell	-0.025 (-0.083, 0.025)	-0.088 (-0.137, -0.026)	-0.069 (-0.101, -0.015)	-0.045 (-0.121, 0.046)	-0.095 (-0.152, -0.002)	-0.114 (-0.169, -0.080)	-0.048 (-0.099, -0.011)	-0.047 (-0.087, -0.017)	-0.010 (-0.021, -0.001)
*M. leucantha* sclerenchyma fiber	-0.078 (-0.100, -0.050)	-0.076 (-0.097, -0.049)	-0.001 (-0.013, 0.010)	-0.080 (-0.110, -0.045)	-0.0782 (-0.108, -0.046)	-0.003 (-0.019, 0.008)	-0.074 (-0.091, -0.049)	-0.076 (-0.092, -0.051)	-0.000 (-0.003, 0.002)
*P. sylvestris* latewood	-0.087 (-0.126, -0.044)	-0.088 (-0.124, -0.044)	-0.004 (-0.021, 0.017)	-0.093 (-0.128, -0.047)	-0.092 (-0.129, -0.042)	-0.001 (-0.021, 0.017)	-0.080 (-0.126, -0.042)	-0.081 (-0.125, -0.048)	-0.001 (-0.006, 0.004)

A detailed description of the sample sizes can be found in **Supplementary Table S1**.

For the *H. crepitans* fruit, *M. leucantha* sclerenchyma fiber and *P. sylvestris* latewood samples, the median strain along the x- and y-axis was estimated by all three approaches in the range of -7.4% to -9.3%, while the median x-axial strain for the *P. jeffreyi* pine cone sclereid cells was estimated lower but with a larger interquartile range by all three approaches [Avizo™: -2.5% (-8.3%, 2.5%), elastix: -4.5% (-12.1%, 4.6%), MBS-3D-OptFlow: -4.8% (-9.9%, -1.1%)]. The median strain along the z-axis is close to zero for all samples except for the *P. jeffreyi* pine cone sclereid cells, where it is comparably closer to the mean strains along the x- and y-axis. Here, elastix estimated the lowest median z-strain (highest shrinkage) at -11.4% (-16.6%, -8.0%), while MBS-3D-OptFlow estimated the highest median z-strain (lowest shrinkage) at -1.0% (-2.1%, -0.1%).

A detailed volume rendering of the axial strain fields demonstrates comparable outcomes when the approaches are considered collectively (**Figure 5A**). The results from Avizo™ and MBS-3D-OptFlow revealed greater inconsistency in the z-strain values at the extremities of the z-stack than the results from elastix, with lower values observed at the lower end and higher values at the upper end (**Figures 5C–E**). In contrast, the elastix and MBS-3D-OptFlow results exhibited strain peaks in close proximity to the pits, which were not discernible in the Avizo™ results (**Figure 5F**).

### 3.3 Evaluation of DVC methods - manufactured solution

**Figure 6:**
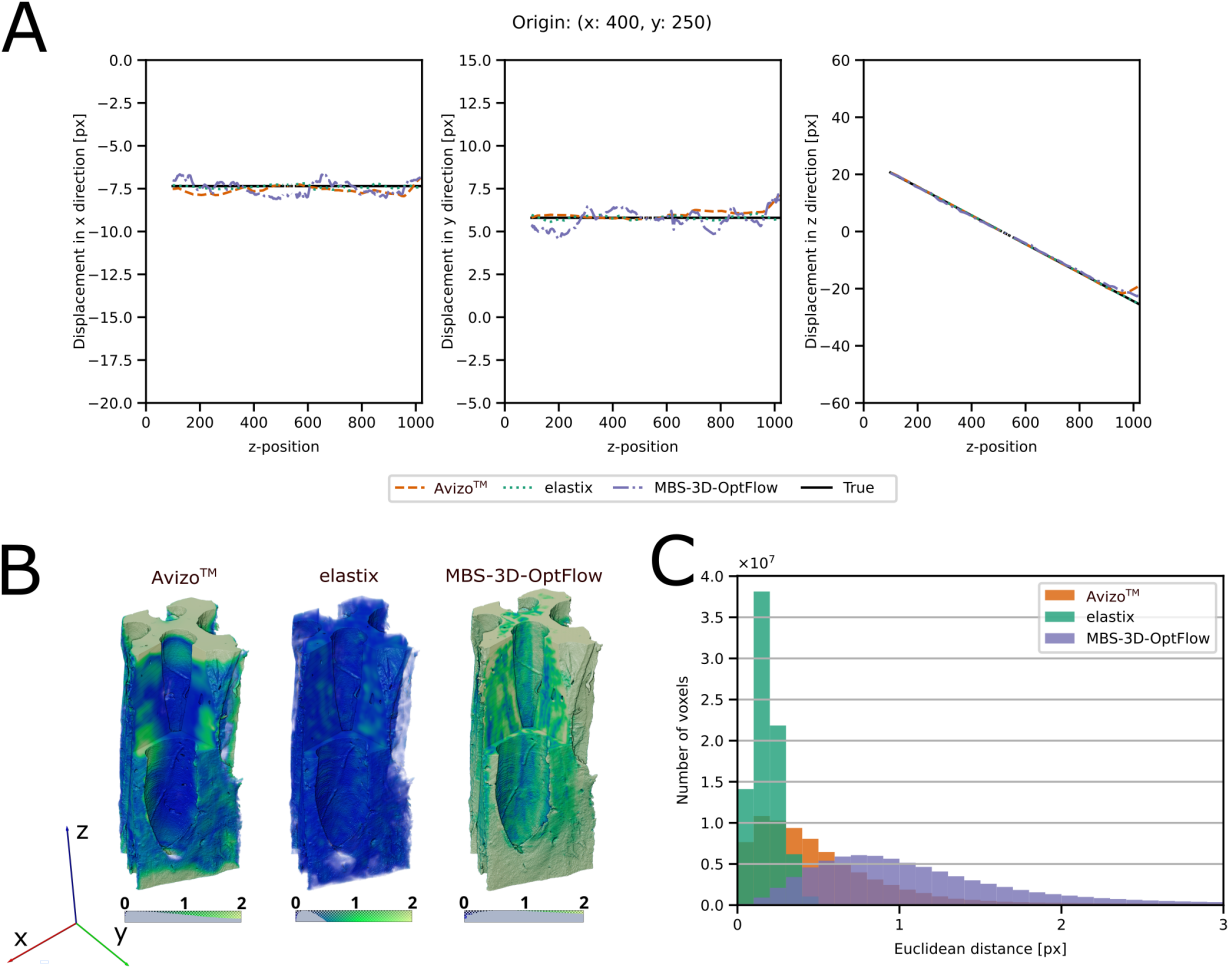
Evaluation of the DVC results of the *Hura crepitans* sample with a linear control. **(A)** Comparison between the x-, y- and z-components of the ground truth and the DVC results for a column of voxels along the z-axis, originating from x = 400 and y = 250. True field: black and solid, elastix: green and dotted, Avizo™: orange and dashed, MBS-3D-OptFlow: violet and dash-dotted. **(B)** Volume rendering of the Euclidean distance field between the true and DVC estimated displacement field. **(C)** Histogram of the Euclidean distance between the true displacement field and the DVC estimated displacement field at each voxel position. A lower Euclidean distance indicates that the DVC tool estimated the displacement field with higher precision. Avizo™: orange, elastix: green, MBS-3D-OptFlow: violet.

**Figure 7:**
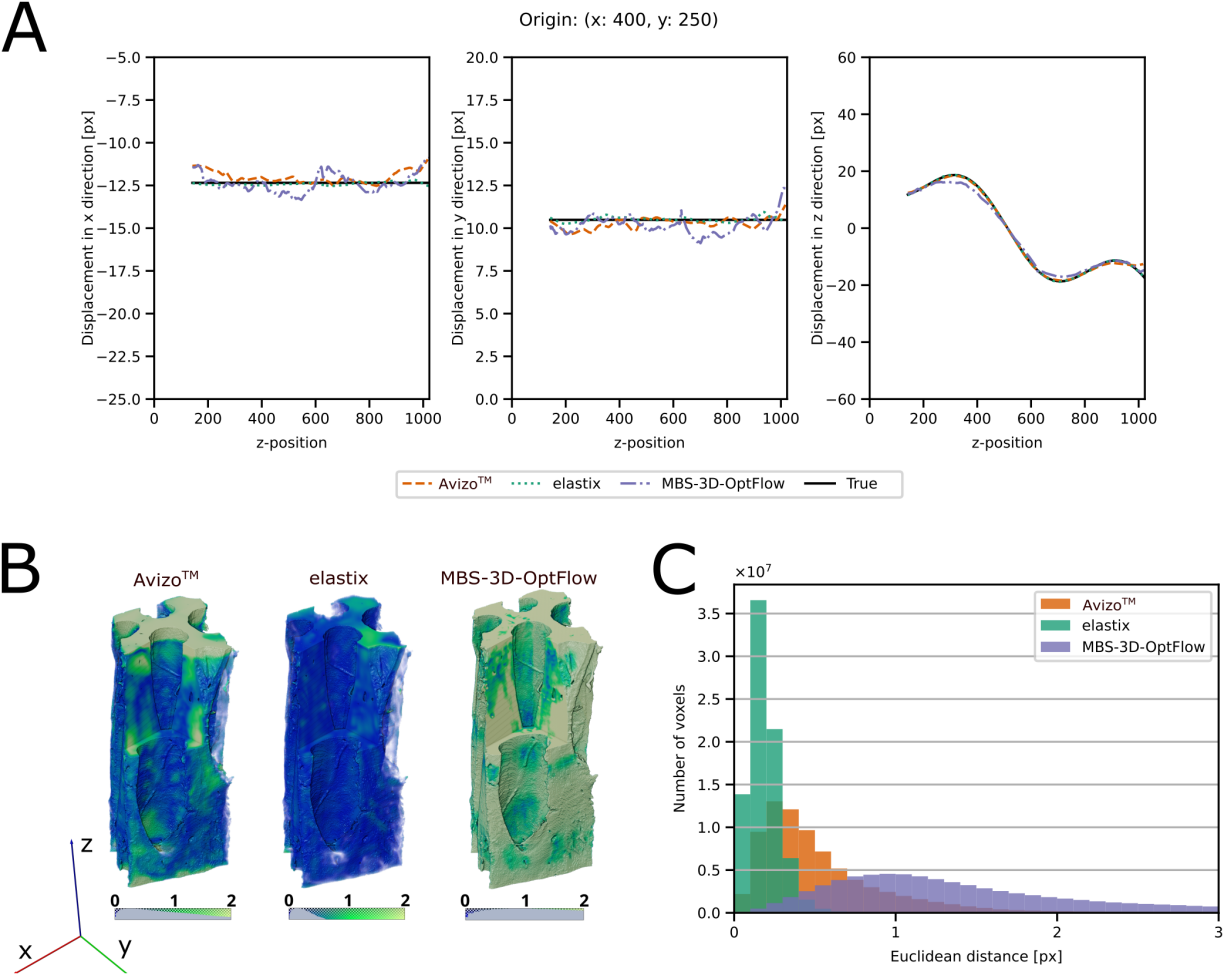
Evaluation of the DVC results of the *Hura crepitans* sample with a sinusoidal control. **(A)** Comparison between the x-, y- and z-components of the ground truth and the DVC results of one column of voxels along the z-axis originating from x = 400 and y = 250. True field: black and solid, elastix: green and dotted, Avizo™: orange and dashed, MBS-3D-OptFlow: violet and dash-dotted. **(B)** Volume rendering of the Euclidean distance field between the true and DVC estimated displacement field. **(C)** Histogram of the Euclidean distance between the true displacement field and the DVC estimated displacement field at each voxel position. A lower Euclidean distance indicates that the DVC tool estimated the displacement field with higher precision. Avizo™: orange, elastix: green, MBS-3D-OptFlow: violet.

The DVC approaches were compared by using a manufactured solution with a known displacement field (**Figures 6**, **7**). The accuracy of the results was estimated by calculating the Euclidean distance between the true displacement field and the displacement field estimated by DVC. **Table 2** provides a summary of the results and detailed visualizations of the pine cone scale sample, the *M. leucantha* sclerenchyma fibers and the *P. sylvestris* latewood are available in **Supplementary Figures S5**-**S10**. For all datasets, elastix achieved a sub-voxel accuracy, with the lowest Euclidean distance for the *H. crepitans* fruit. For the linear and the sinusoidal control of the *H. crepitans* fruit and the *M. leucantha* sclerenchyma fiber, Avizo™ achieved sub-voxel accuracy (**Table 2**). MBS-3D-OptFlow achieved a sub-voxel accuracy for both controls of the pine cone scale sample. However, with a median of 4.03 voxel, the MBS-3D-OptFlow analysis of the sinusoidal deformation of the *P. sylvestris* latewood resulted in the highest Euclidean distances across all calculations. In the course of analyzing the results, it was also noted that in particular Avizo™ and MBS-3D-OptFlow exhibited a tendency to encounter difficulties at the upper and lower extremities of the z-stack.

**Table 2:** A comparison of the median Euclidean distance between the true and DVC-estimated displacement fields across the sample bulk of the wet-state image.

Sample	Linear control			Sinusoidal control		
	Avizo™	Elastix	MBS-3D-Opt-Flow	Avizo™	Elastix	MBS-3D-Opt-Flow
*H. crepitans* fruit	0.43 (0.22, 0.78)	0.17 (0.12, 0.23)	1.01 (0.66, 1.52)	0.43 (0.27, 0.74)	0.17 (0.12, 0.24)	1.33 (0.86, 2.19)
*P. jeffreyi* pine cone scale sclereid cell	1.36 (0.63, 2.34)	0.27 (0.16, 0.41)	0.38 (0.16 1.47)	2.73 (1.34, 5.47)	0.36 (0.23, 0.56)	0.55 (0.31, 1.46)
*M. leucantha* sclerenchyma fiber	0.38 (0.19, 0.81)	0.20 (0.13, 0.28)	0.93 (0.51, 1.67)	0.95 (0.54, 1.65)	0.25 (0.16, 0.37)	1.40 (0.82, 2.65)
*P. sylvestris* latewood	1.07 (1.00, 1.31)	0.16 (0.10, 0.23)	2.68 (1.48, 5.46)	1.86 (0.79, 3.89)	0.25 (0.17, 0.39)	4.03 (2.04 7.82)

The 25th and 75th percentiles are indicated in brackets. A detailed description of the sample sizes can be found in **Supplementary Table S2**.

## 4 Discussion

Not only did the different DVC analysis approaches deliver comparable results, but these results were also within the expected range for the hygroscopic shrinkage of the lignocellulosic tissues studied (Dawson et al., 1997; Thybring and Fredriksson, 2023). As the sample dimensions are in the range of micrometers, the poroelastic timescale was expected to be in the range of seconds to minutes (Poppinga et al., 2018). Since the samples had no further observable movement after three minutes, it can be assumed that equilibrium was reached during the conditioning period. As the selected climate levels cover the upper and lower end of the hygroscopic range, we can assume that our results represent the maximum deformation and strain of the samples in the measurable hygroscopic range at P05. But as the history of the sample remains unknown, we cannot assess the influence of sorption hysteresis (Thybring and Fredriksson, 2023). However, the actual moisture content of the cell wall was not measured during scanning. Additionally, the effect on the moisture content in respect to the heating of the sample through the X-ray beam was not considered.

Upon examination of the strains along the x- and y-axes, it is evident that the highest strain values are typically observed in cell wall regions oriented perpendicular to the axis under consideration. This observation was made in all four samples and in all DVC results, indicating that in general the cell wall shrinkage is higher along the cell wall thickness than parallel to the cell wall in the transversal plane. Due to the limited number of locations within the sample volumes where cell walls were oriented perpendicular to the z-axis, no comparable observation could be made in the x-z- or y-z-plane. This observation coincides with that of anisotropy in the transversal plane at the cell wall level, as previously described by Rafsanjani et al. (2014). Their described large transverse anisotropy in swelling/shrinkage of "micropillars" within the S2 layer could therefore also be observed within the cell wall when observing a bundle of neighboring cells. The origin of this swelling/shrinkage behavior has not yet been clarified, as Arzola-Villegas et al. (2019) discuss in detail.

A number of factors must be considered when using DVC to analyze hygroscopic swelling and shrinkage at the microscopic level. The accuracy of DVC depends on the number of internal cell wall features. Moreover, it is further compromised by changes in the grey value distribution caused by the absorption and desorption of water in the cell walls. Therefore, it is essential to select an appropriate correlation metric that can partially compensate for these effects. Additionally, drying-induced structural changes in the samples may lead to crack formation or delamination, resulting in high local deformations in the surrounding regions. To counteract this, it is preferable to use a composition of cells, like those from the *H. crepitans* fruit, *M. leucantha* sclerenchyma fiber and pine latewood samples, rather than single, partially cut cells, as was the case for the pine cone scale sample. Additionally, this can reduce the volumetric fraction and the number of events within the sample volume.

The pine cone scale sample consisted to a large extent of a single, partially cut cell wall. Possibly, due to this sample shape, it proved to be a challenge for the analysis, as evidenced by the differing results and the comparatively high standard deviation in the overall strain distribution when analyzing the acquired data. However, the trend of the mean x-, y-, and z-axial strain values indicate a lower expansion along the x- and y-axis and a larger expansion along the z-axis compared to the other samples. This also corresponds to the previously described higher longitudinal elongation of the pine cone scale sclereid layer, that, due to the high microfibril angle in the cell walls (Dawson et al., 1997), drives the hygroscopic bending movement of pine cone scales. In addition, the linear and sinusoidal control of Avizo™ and elastix showed the greatest inaccuracy with the pine cone scale sample, while MBS-3D-OptFlow was able to achieve sub-voxel accuracy. The lack of unique internal structures (e.g. pits, undulations, cracks, slits etc.), which are important landmarks for a volume correlation, and the general shape of the sample can impede precise evaluation. In contrast, specimens like the one of *H. crepitans* are particularly suitable for analysis as the abundance of internal structural features and the sample composition of multiple cells aid the DVC analysis, improving the accuracy of detecting hygroscopic deformation at the global and local level.

We used two artificially deformed datasets to evaluate the accuracy of DVC, as the precision of the deformation can only be estimated without knowing the ground truth. The linear control resembles an orthotropic shrinkage, and the sinusoidal control resembles an inhomogeneous anisotropic shrinkage of the sample. When checking the Euclidean distance between the DVC estimated displacement fields and the artificially created ground truth, all three approaches were able to achieve sub-voxel accuracy for both controls of at least one sample. The results obtained may also be employed to evaluate potential constraints associated with the utilization of DVC for the analysis of hygroscopic deformation. Although some scenarios, such as cracking or reconstruction artefacts, cannot be easily mimicked artificially, the general estimation of hygroscopic shrinkage at global and local scales can be assumed to be comparable. While it is beyond the scope of this study to investigate in detail the effect of each adjustable parameter of the DVC approaches, it is possible that more accurate results could be obtained by fine-tuning the parameters. Upon comparing the DVC approaches in terms of accuracy, elastix as an accessible open-source 3D image registration toolkit proves to be equally accurate or better in quantifying the hygroscopic deformation of lignocellulosic tissue samples at the cellular level. However, the reason why elastix performs so well on our artificial datasets may also be due to the fact that the initial affine transformation can easily depict an orthotropic shrinkage. Further, its free-form transformation is based on B-splines, which allows a good approximation of the smooth sinusoidal deformation.

By comparing the performance between manufactured solutions using MBS-3D-OptFlow, it shows a better result by the linear control than the sinusoidal control, because optical flow methods generally assume a dense and local deformation field, to retain the convexity of energy loss during optimization. Therefore, using the optical flow algorithm to determine the sinusoidal deformation is a highly ill-posed problem. For example, the deformation can project two voxels in the opposite direction of the zero-crossing position into the zero-crossing position of the deformed volume, which would result in an incorrect overlay of textures from three different voxels on the deformed volume. Also, the multi-scale image pyramid approach of MBS-3D-OptFlow tends to estimate the shortest deformation path instead of longer deformations, which can also increase its difficulty in handling sinusoidal fields. This difficulty in correctly estimating sinusoidal deformations agrees with the findings of Wong et al. (2024), that MBS-3D-OptFlow could not resemble star field-like deformations, which were characterized by sinusoidal deformations in one spatial direction. In prior works, optical flow methods have been broadly studied on linear translational or rotational motion fields (Baker et al., 2011). However, to the best of the authors’ knowledge, optical flow methods were rarely tested or discussed for periodic flow fields. This can be an interesting perspective for future work, to enable a broader applicability of optical flow-based algorithms.

The results demonstrate that MBS-3D-OptFlow tends to be robust to abrupt changes even in the inner structures without rich texture, while it can still estimate displacement fields with sub-voxel accuracy for samples with rich structural information. However, MBS-3D-OptFlow shows its limitation on large and smooth displacement fields, since it was mainly developed to reveal morphological relationships in a more local oriented perspective (Schmelzle et al., 2021; Bruns et al., 2023). Generally, variational methods such as MBS-3D-OptFlow can be optimized by individually fine-tuning its parameters according to datasets. Although the experimental results show a certain level of generalizability among synthetic and realistic data, deep learning methods such as the neural network VolRAFT (Wong et al., 2024) can provide opportunities to reduce the expertise skill required for optimizing the DVC analysis.

In general, DVC will prove to be an important tool as the fields of wood science, biomechanics and biomimetics delve deeper into hygroscopic swelling and shrinking as they aim to better understand how, for example, macroscopic movements are enabled. Our analysis at the cellular level reveals the potential of Avizo™, elastix and MBS-3D-OptFlow to describe hygroscopic deformations with a sub-voxel accuracy (**Table 2**). The change in greyscale distribution due to water desorption appears to be manageable by choosing an appropriate correlation metric. Still, the formation of structural defects such as cracks or delamination of sample elements due to drying can compromise the accuracy in the immediate vicinity of the defect. In terms of use cases, Avizo™ is easy to use with a graphical user interface and built-in tools to calculate and visualize the resulting strain field (**Table 3**). Both elastix and MBS-3D-OptFlow are command line tools with no graphical user interface or built-in strain calculation and visualization (**Table 3**). Upon comparing Avizo™, elastix and MBS-3D-OptFlow using manufactured solutions, the accuracy of the software is comparable. While elastix seems to work well with smooth deformations, due to its combined affine and B-spline transformation-based approach, Avizo™ and MBS-3D-OptFlow may outperform in the case of crack formation and more abrupt changes. Further investigating the impact of the selected parameters, such as the edge length of the tetrahedral grids of Avizo™, or the control grid size of elastix, and the integration of a model for hygroscopic swelling and shrinkage as a regularization across the grids, may contribute to an even more precise and detailed estimation of the hygroscopic deformation.

**Table 3:** Comparison of the tools used to perform DVC on lignocellulosic tissues.

Parameter	Avizo™	Elastix	MBS-3D-OptFlow
Interface	Graphical user interface	Command line interface	Command line interface
Features	All-in-one software: Pre-processing possible in software Residuals and displacement increment for immediate control of the fit Visualization of results possible in software	C++ based command line tool with optional python interface Pre-processing (pre-registration) available in repository Additional software needed for pre-processing analysis of the displacement fields	C++ and Cuda based DVC tool Pre-processing (pre-registration) and strain export available in repository Additional software needed for pre-processing or analysis of the displacement fields
Workflow DVC	1) Local DVC (optional): independent registration of sub-volumes 2) Global DVC: mesh-based registration	1) Affine registration 2) B-Spline registration 3) Retrieval of displacement field based on transformation parameters	1) Local-global approach: local patch alignment regularized by the global smoothness
Customizability	Limited control over the hyperparameters (e.g., mesh size and regularization term) Extendable with Python API	Full accessibility and control over all hyperparameters by default	Full accessibility and control over all hyperparameters by default
Accuracy	Sub-voxel accuracy	Sub-voxel accuracy	Sub-voxel accuracy
Applied test cases	Compare **Table 2**	Compare **Table 2**	Compare **Table 2**
Licensing	Commercial Avizo™ with DVC extension	Freeware Open source (Apache-2.0 license)	Freeware Open source (MIT license)

## Data Availability

The datasets presented in this study can be found in online repositories. All primary data (CT-reconstructions as .tiff) to support the findings of this study are openly available under https://doi.org/10.25592/uhhfdm.16522 (ZFDM repository, Center for sustainable research data management, University of Hamburg).
