# Correction: Quantifying hygroscopic deformation in lignocellulosic tissues: a digital volume correlation tool comparison

**DOI:** 10.3389/fpls.2026.1924467

**Published:** 2026-07-08

**Authors:** Kim Ulrich, Fabian Scheckenbach, Tak Ming Wong, Tom Masselter, Silja Flenner, Anaclara Visconti, Martin Nopens, Andreas Krause, Sergej Kaschuro, Jakob Benedikt Mietner, Thomas Speck, Imke Greving, Berit Zeller-Plumhoff, Linnea Hesse

**Affiliations:** 1 Plant Biomechanics Group @ Botanic Garden, University of Freiburg, Freiburg im Breisgau, Germany; 2 Cluster of Excellence livMatS @ FIT—Freiburg Center for Interactive Materials and Bioinspired Technologies, University of Freiburg, Freiburg im Breisgau, Germany; 3 Biomimetics Group, Institute of Wood Sciences, University of Hamburg, Hamburg, Germany; 4 Institute of Metallic Biomaterials, Helmholtz-Zentrum Hereon, Geesthacht, Germany; 5 Institute of Materials Physics, Helmholtz-Zentrum Hereon, Geesthacht, Germany; 6 Research Area Landscape Functioning, Leibniz Center for Agricultural Landscape Research (ZALF) e.V., Müncheberg, Germany; 7 Thünen Institute of Wood Research, Thünen Institute, Hamburg, Germany; 8 Data-driven Analysis and Design of Materials, Faculty of Mechanical Engineering and Marine Technologies, University of Rostock, Rostock, Germany

**Keywords:** biomechanics, wood science, lignocellulose, DVC, hygroscopy, computed tomography

In the **Acknowledgments** statement, the following sentence was missing “This work was supported by the Deutsche Forschungsgemeinschaft (DFG, German Research Foundation) through the Cluster of Excellence EXC3120 BlueMat: Water-Driven Materials. We acknowledge Helmholtz-Zentrum Hereon and DESY for the provision of the experimental infrastructure and the provision of beamtime for proposal(s) I-20230453 and I-20221356.”.

The original version of this article has been updated.

