# Supplementary material for: Quantifying hygroscopic deformation in lignocellulosic tissues: a digital volume correlation tool comparison
Source: Front Plant Sci. 2025 Aug 18;16:1572745. doi: 10.3389/fpls.2025.1572745 (PMC12399559; doi:10.3389/fpls.2025.1572745)
Supplement: Supplementary file 1 [file DataSheet1.docx]

Supplementary Material

**Table S1:** Number of voxels across the masked sample bulk evaluated using DVC on the original, naturally deformed data.

| sample | Avizo™ | elastix | MBS-3D-Opt-Flow |
| --- | --- | --- | --- |
| *H. crepitans* fruit | 80031968 | 80401760 | 80401760 |
| *P. jeffreyi* pine cone sclereid cell | 177026210 | 177628537 | 177628537 |
| *M. leucantha* sclerenchyma fiber | 595380121 | 598933728 | 598933728 |
| *P. sylvestris* latewood | 473956045 | 475684826 | 475684826 |

**Table S2:** Number of voxels across the masked sample bulk evaluated using DVC on the artificially deformed data.

| sample | Linear control | Sinusoidal control |
| --- | --- | --- |
| *H. crepitans* fruit | 81467792 | 80667855 |
| *P. jeffreyi* pine cone sclereid cell | 185292858 | 181955438 |
| *M. leucantha* sclerenchyma fiber | 736354348 | 739368168 |
| *P. sylvestris* latewood | 479442715 | 473356510 |

**
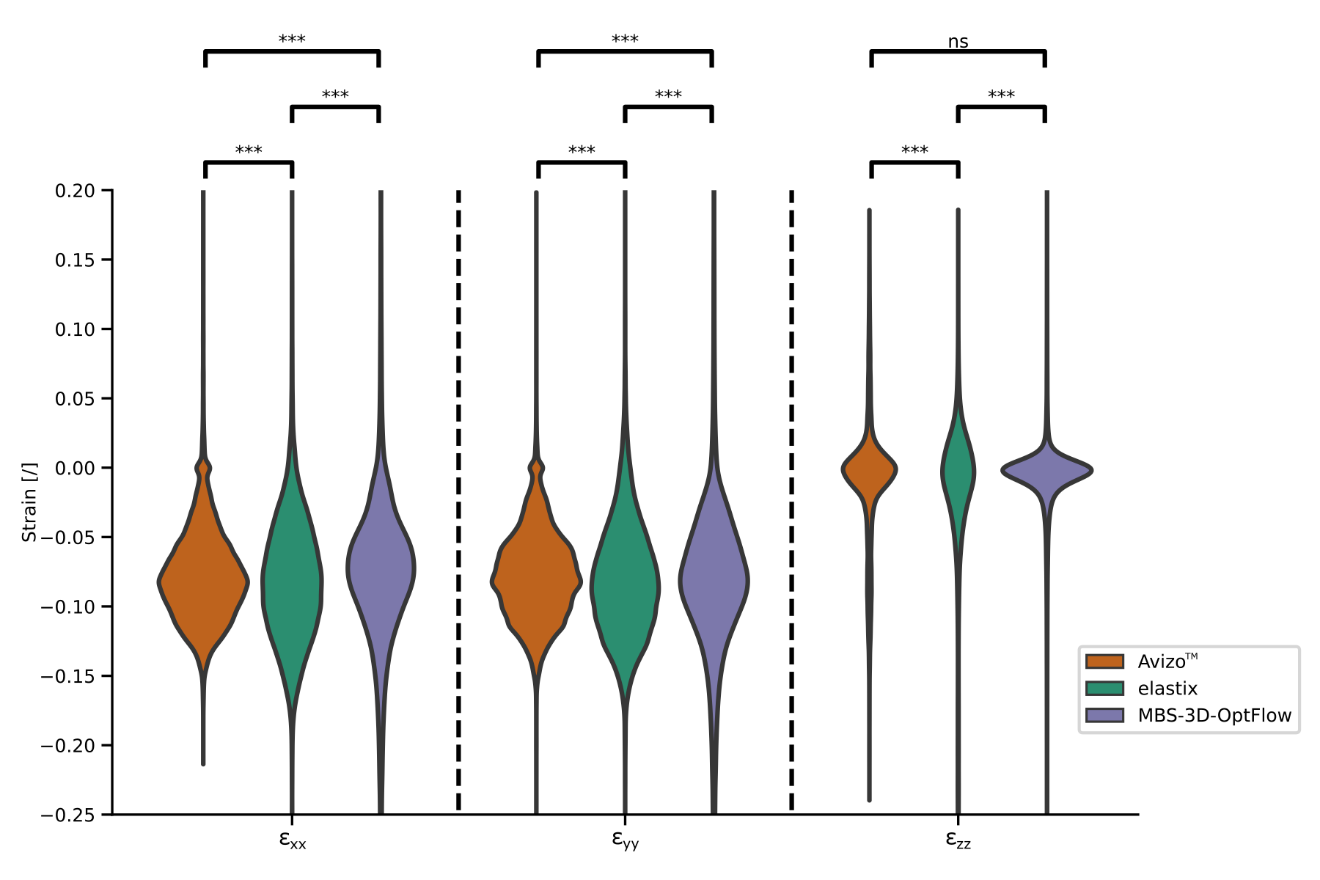
Figure S1:** Axial strain of the *Hura crepitans* fruit sample using Avizo™, elastix and MBS-3D-OptFlow. Significant differences (p < 0.001; Mann-Whitney U test) in all but one paring, with the absolute effect size (difference of the mean) being smaller than 0.006 for all three axial strains.


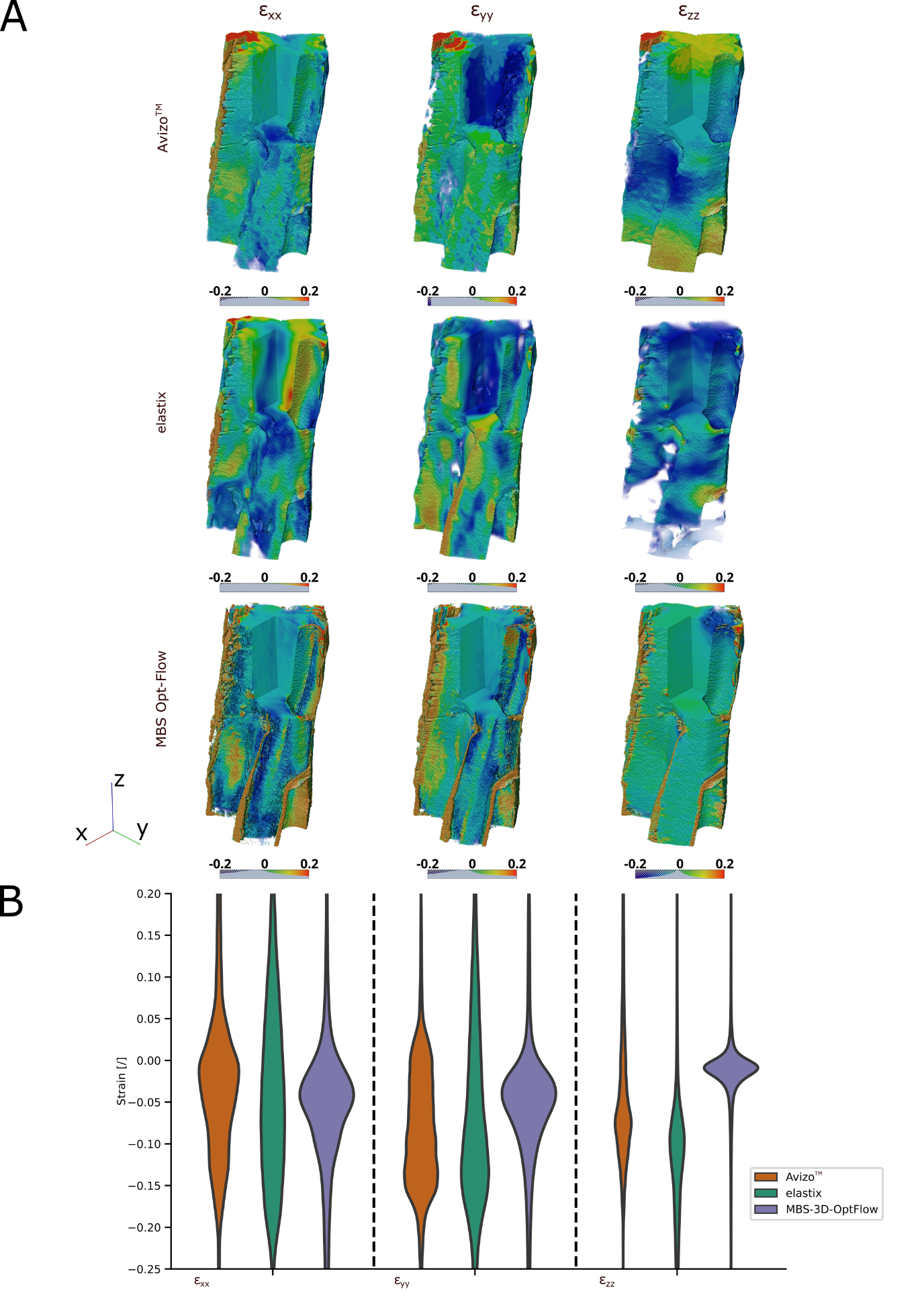


**Figure S2:** 3D visualisation and violin plot of the DVC analysis of the *Pinus jeffreyi* sclereid cell of the pine cone scale using Avizo™, elastix and MBS-3D-OptFlow. (A) Volume rendering of the masked x-, y-, and z-axial components of the Green-Lagrangian strain with a visual cropping of the front corner to display interior strains. Each image row distinguishes one of the three software used. In case of Avizo, the upper- and lowermost slices, which were not covered by the mesh, and thus contain strain values of zero are cut off. (B) Distribution of the axial strains across a randomised subsample of 100.000 voxels of the bulk of the reference image sample.


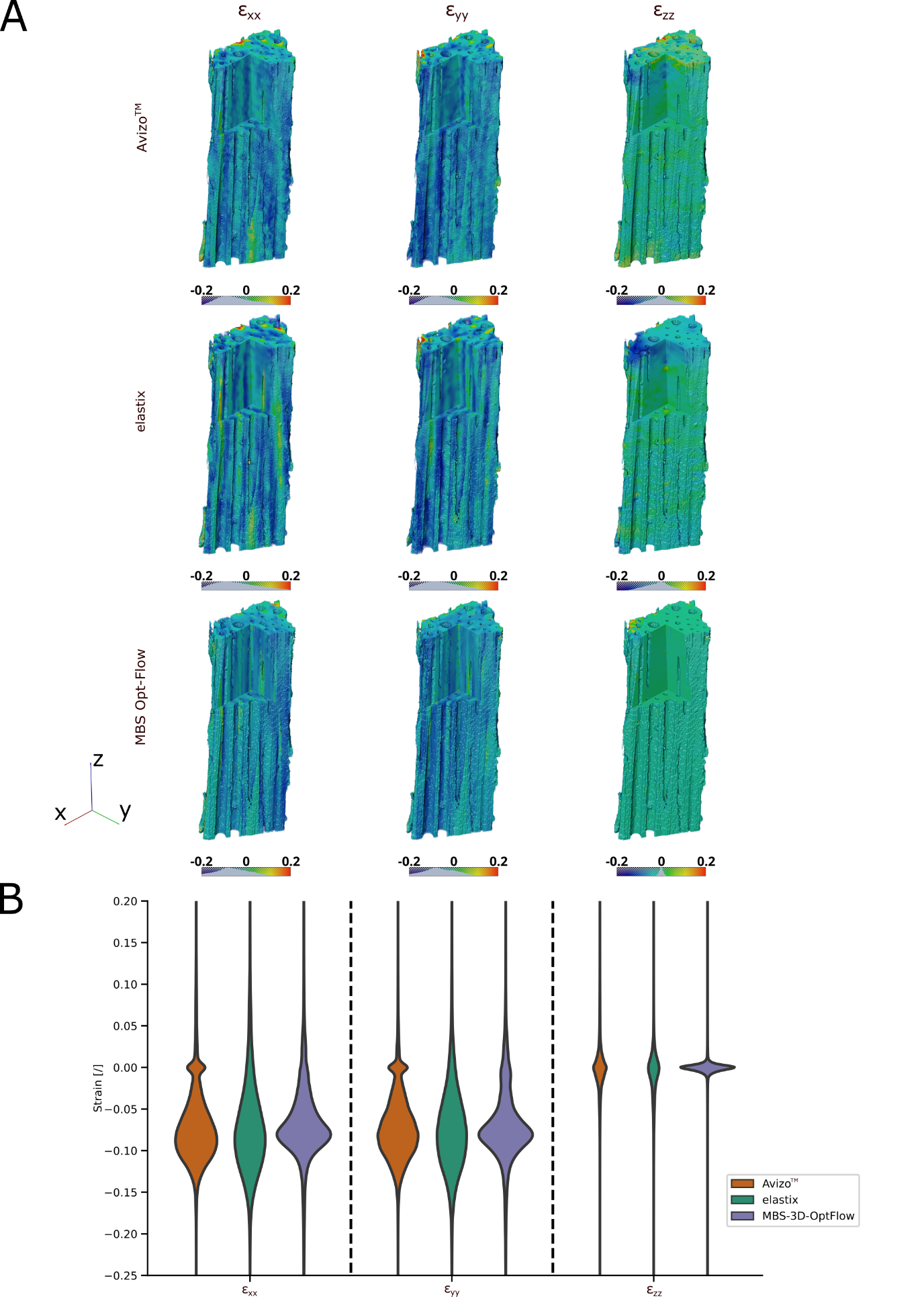


**Figure S3:** 3D visualisation and violin plot of the DVC analysis of the *Marantochloa leucantha* sclerenchyma fiber sheath sample using Avizo™, elastix and MBS-3D-OptFlow. (A) Volume rendering of the masked x-, y-, and z-axial components of the Green-Lagrangian strain with a visual cropping of the front corner to display interior strains. Each image row distinguishes one of the three software used. In case of Avizo, the upper- and lowermost slices, which were not covered by the mesh, and thus contain strain values of zero are cut off. (B) Distribution of the axial strains across a randomised subsample of 100.000 voxels of the bulk of the reference image sample.


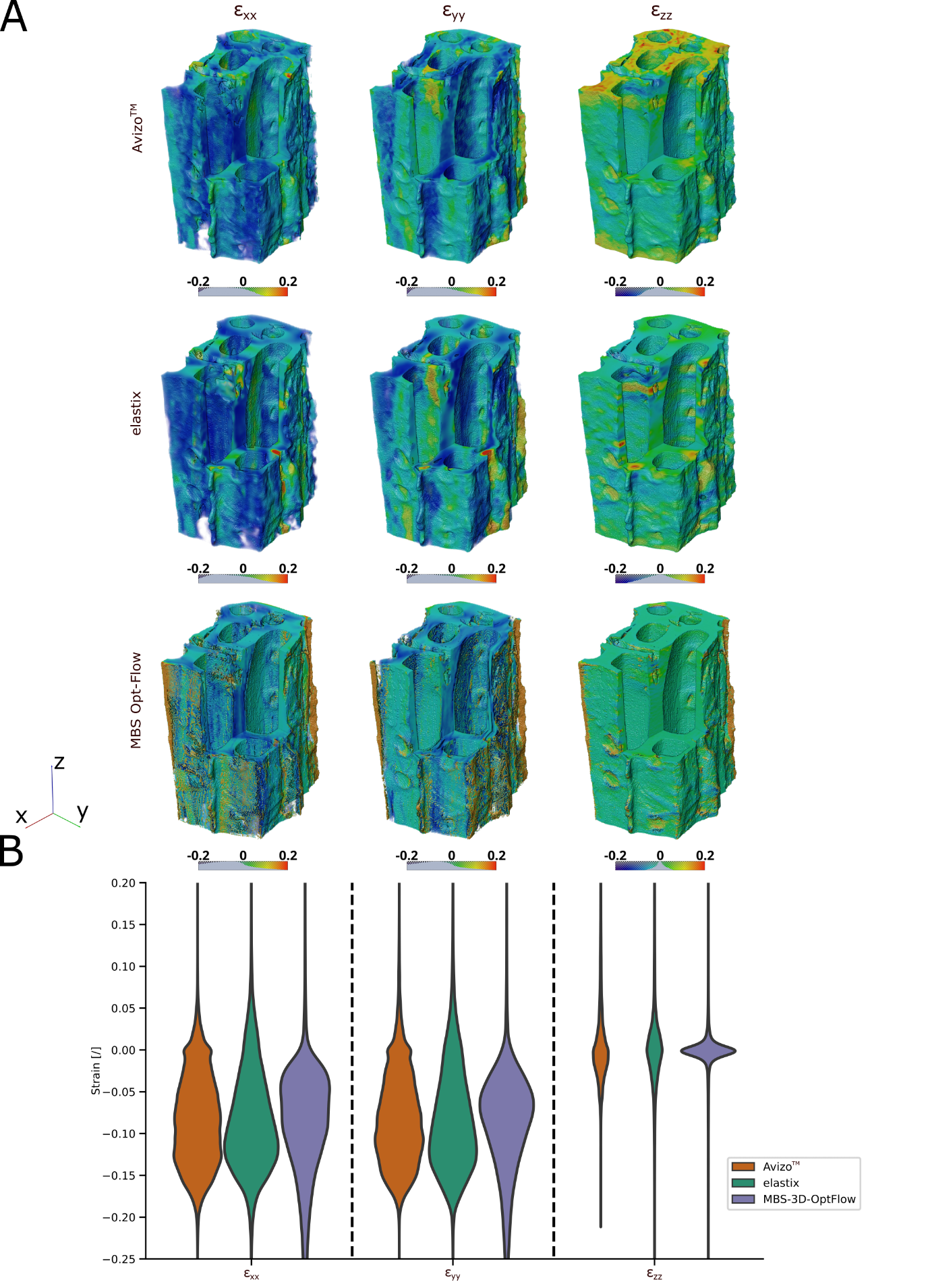
 **Figure S4:** 3D visualisation and violin plot of the DVC analysis of the *Pinus sylvestris* latewood sample using Avizo™, elastix and MBS-3D-OptFlow. (A) Volume rendering of the masked x-, y-, and z-axial components of the Green-Lagrangian strain with a visual cropping of the front corner to display interior strains. Each image row distinguishes one of the three software used. In case of Avizo™, the upper- and lowermost slices, which were not covered by the mesh, and thus contain strain values of zero are cut off. (B) Distribution of the axial strains across a randomised subsample of 100.000 voxels of the bulk of the reference image sample.


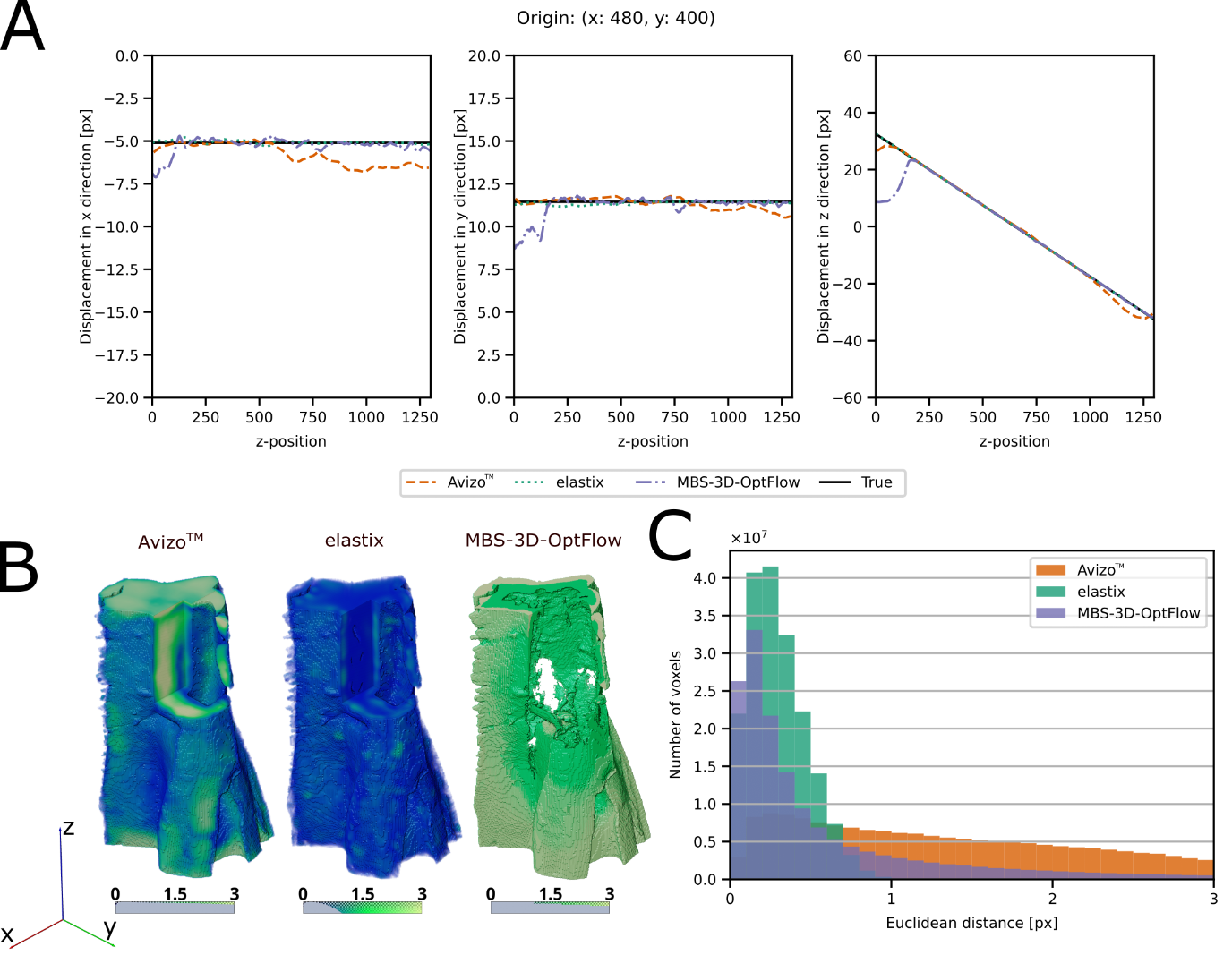


**Figure S5:** Evaluation of the DVC results of the *Pinus jeffreyi* sclereid cell of the pine cone scale with a linear control. (A) Comparison between the x-, y- and z-components of the ground truth and the DVC results for a column of voxels along the z-axis, originating from x=480 and y=400. True field: black and solid, elastix: green and dotted, Avizo™: orange and dashed, MBS-3D-OptFlow: violet and dash-dotted. (B) Volume rendering of the Euclidean distance field between the true and DVC estimated displacement field. (C) Histogram of the Euclidean distance between the true displacement field and the DVC estimated displacement field at each voxel position. A lower Euclidean distance indicates that the DVC tool estimated the displacement field with higher precision. Avizo™: orange, elastix: green, MBS-3D-OptFlow: violet.


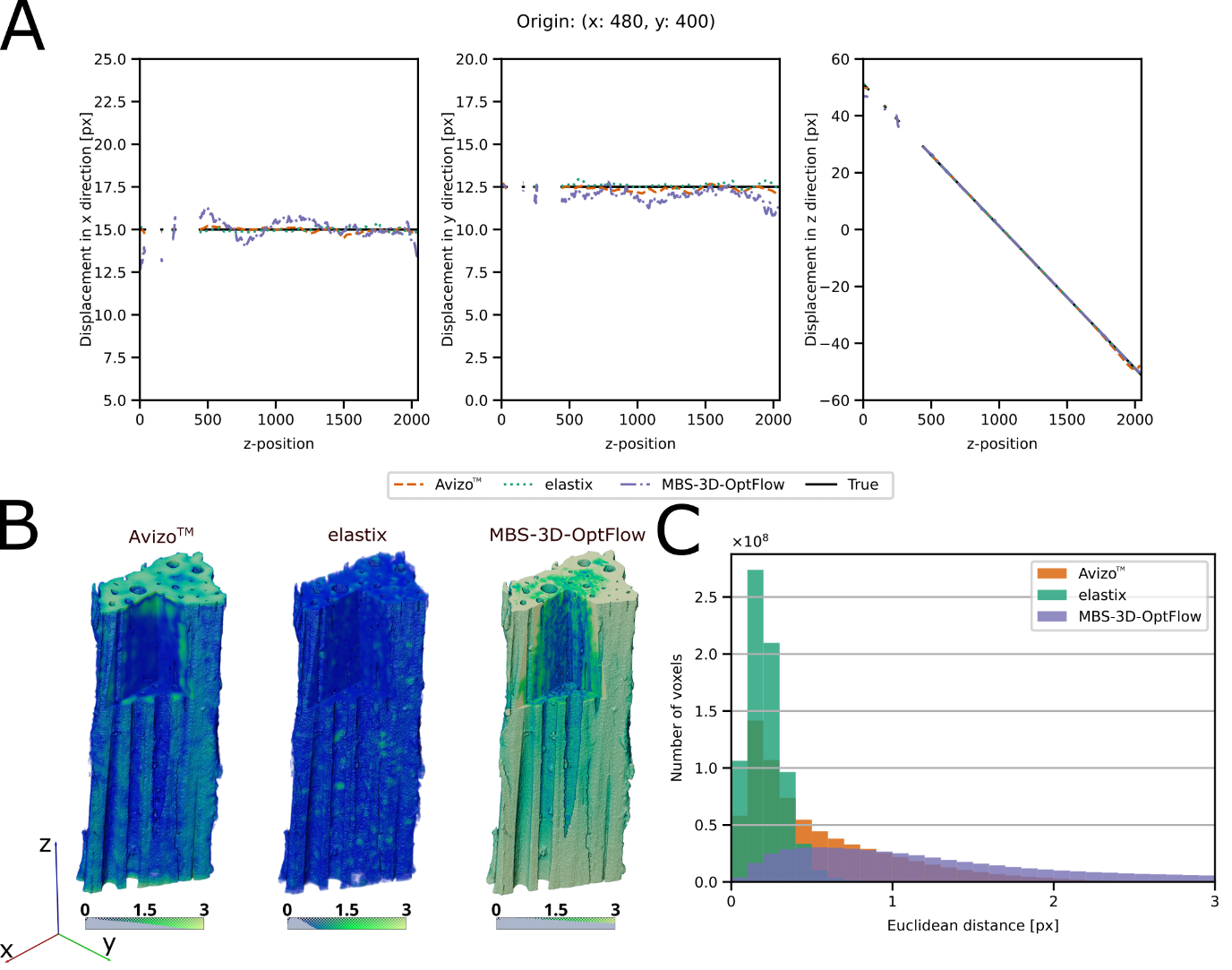


**Figure S6:** Evaluation of the DVC results of the *Marantochloa leucantha* sclerenchyma fibre sheath sample with a linear control. (A) Comparison between the x-, y- and z-components of the ground truth and the DVC results for a column of voxels along the z-axis, originating from x=480 and y=400. True field: black and solid, elastix: green and dotted, Avizo™: orange and dashed, MBS-3D-OptFlow: violet and dash-dotted. (B) Volume rendering of the Euclidean distance field between the true and DVC estimated displacement field. (C) Histogram of the Euclidean distance between the true displacement field and the DVC estimated displacement field at each voxel position. A lower Euclidean distance indicates that the DVC tool estimated the displacement field with higher precision. Avizo™: orange, elastix: green, MBS-3D-OptFlow: violet.


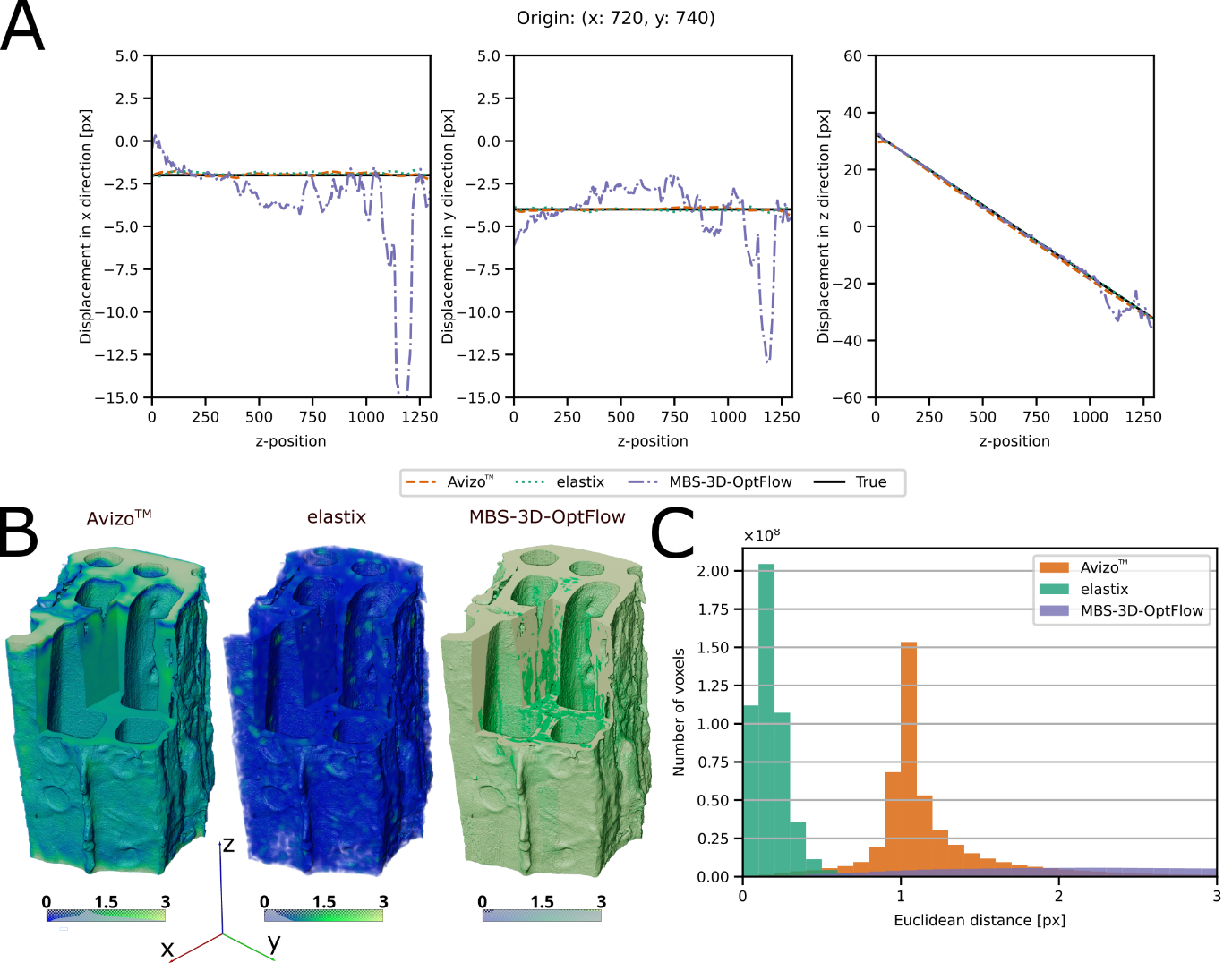


**Figure S7:** Evaluation of the DVC results of the *Pinus sylvestris* latewood sample with a linear control. (A) Comparison between the x-, y- and z-components of the ground truth and the DVC results for a column of voxels along the z-axis, originating from x=720 and y=740. True field: black and solid, elastix: green and dotted, Avizo™: orange and dashed, MBS-3D-OptFlow: violet and dash-dotted. (B) Volume rendering of the Euclidean distance field between the true and DVC estimated displacement field. (C) Histogram of the Euclidean distance between the true displacement field and the DVC estimated displacement field at each voxel position. A lower Euclidean distance indicates that the DVC tool estimated the displacement field with higher precision. Avizo™: orange, elastix: green, MBS-3D-OptFlow: violet.


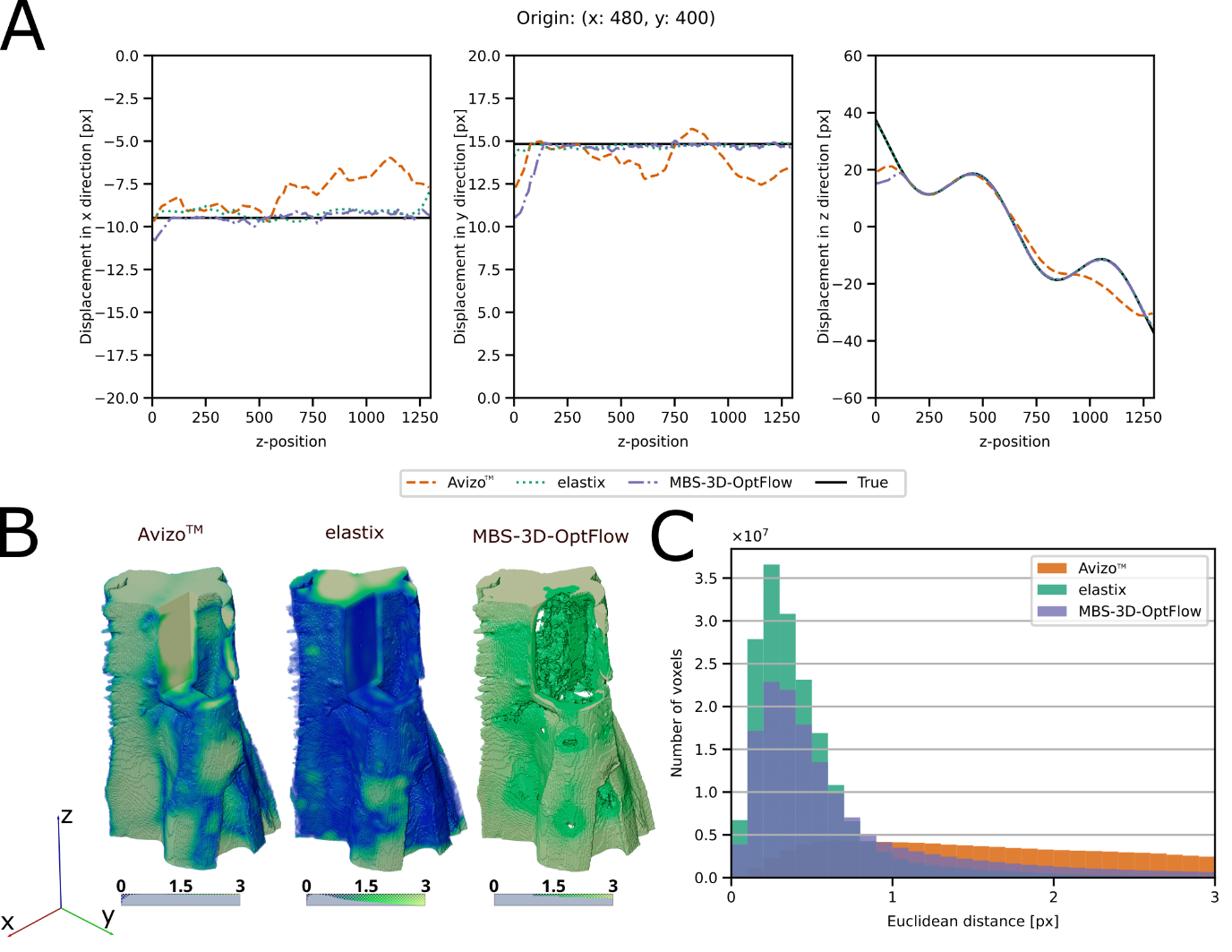


**Figure S8:** Evaluation of the DVC results of the *Pinus jeffreyi* sclereid cell of the pine cone scale with a sinusoidal control. (A) Comparison between the x-, y- and z-components of the ground truth and the DVC results of one column of voxels along the z-axis originating from x=480 and y=400. True field: black and solid, elastix: green and dotted, Avizo™: orange and dashed, MBS-3D-OptFlow: violet and dash-dotted. (B) Volume rendering of the Euclidean distance field between the true and DVC estimated displacement field. (C) Histogram of the Euclidean distance between the true displacement field and the DVC estimated displacement field at each voxel position. A lower Euclidean distance indicates that the DVC tool estimated the displacement field with higher precision. Avizo™: orange, elastix: green, MBS-3D-OptFlow: violet.


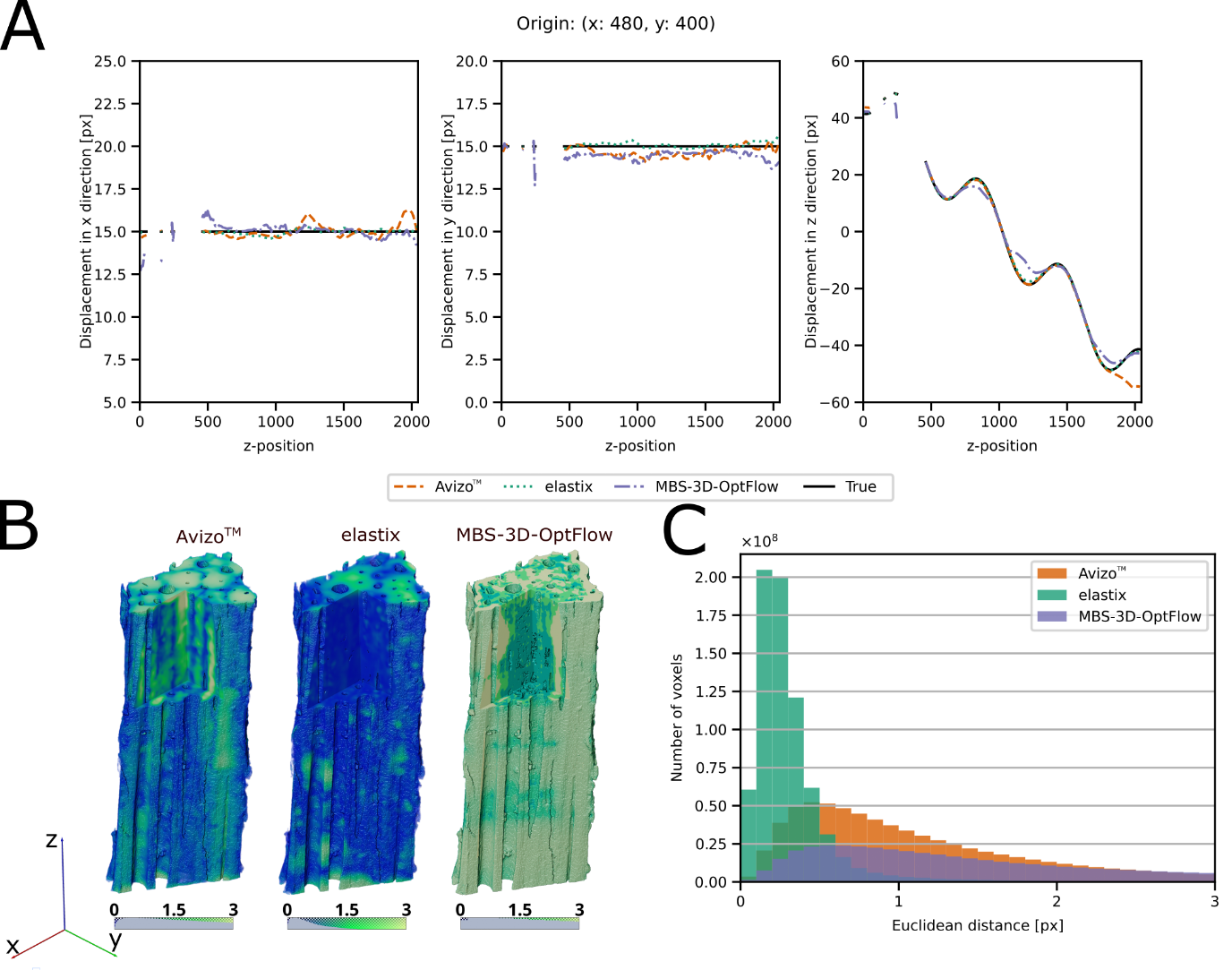
**Figure S9:** Evaluation of the DVC results of the *Marantochloa leucantha* sclerenchyma fibre sheath sample with a sinusoidal control. (A) Comparison between the x-, y- and z-components of the ground truth and the DVC results of one column of voxels along the z-axis originating from x=480 and y=400. True field: black and solid, elastix: green and dotted, Avizo™: orange and dashed, MBS-3D-OptFlow: violet and dash-dotted. (B) Volume rendering of the Euclidean distance field between the true and DVC estimated displacement field. (C) Histogram of the Euclidean distance between the true displacement field and the DVC estimated displacement field at each voxel position. A lower Euclidean distance indicates that the DVC tool estimated the displacement field with higher precision. Avizo™: orange, elastix: green, MBS-3D-OptFlow: violet.


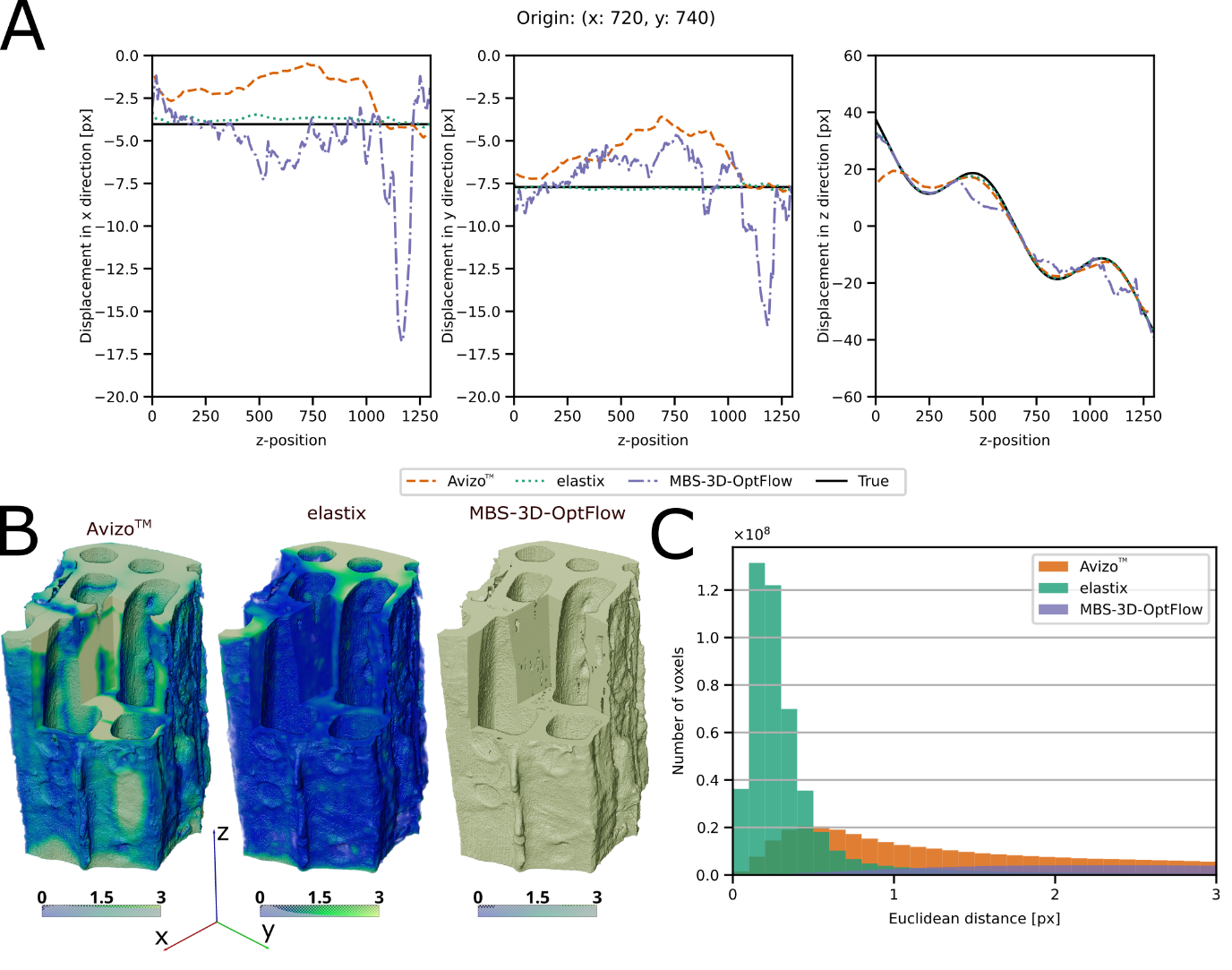


**Figure S10:** Evaluation of the DVC results of the *Pinus sylvestris* latewood sample with a sinusoidal control. (A) Comparison between the x-, y- and z-components of the ground truth and the DVC results of one column of voxels along the z-axis originating from x=720 and y=740. True field: black and solid, elastix: green and dotted, Avizo™: orange and dashed, MBS-3D-OptFlow: violet and dash-dotted. (B) Volume rendering of the Euclidean distance field between the true and DVC estimated displacement field. (C) Histogram of the Euclidean distance between the true displacement field and the DVC estimated displacement field at each voxel position. A lower Euclidean distance indicates that the DVC tool estimated the displacement field with higher precision. Avizo™: orange, elastix: green, MBS-3D-OptFlow: violet.

**Step-by-step instruction for applying DVC on CT images**

Preparation of image data with FIJI
(BEFORE: Check in FIJI if under “Edit”->”Options”->”Input/Output…” the checkmark at “Save TIFF and raw in intel byte order is checked”)

1. Open the reference and deformed images in FIJI (e.g. as an Image Sequence, Figure 1)


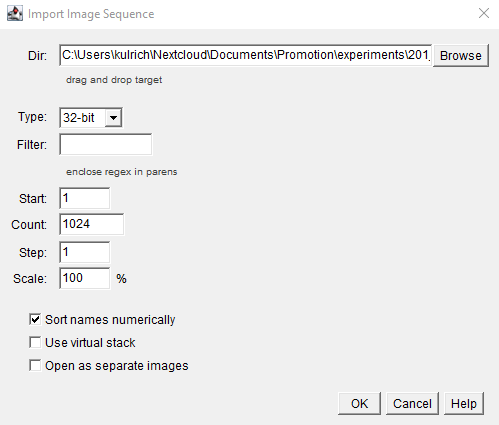


Figure S11: Import Image sequence window.

1. Convert the image bit size to 8-bit under “Image” -> ”Type” -> “8-bit”
   By converting the image bit type you can save storage and computation time but reduce your grayscale values to a total of 256 values.
2. If applicable, crop your image to a Region of Interest (ROI) by using the selection tool. The resulting volume of reference and deformed state should have the same size.
   1. select the ROI in one image.
   2. restore the selection in the second image with CTRL+SHIFT+E (“Edit” -> “Selection” -> “Restore Selection”) and increase the selection based on the second sample image
   3. restore the selection back to the first image.
   4. crop both images to the selection with CTRL+SHIFT+X.
3. Check if there is a big z-shift of your sample visible in the images (e.g. parts of your reference state not visible anymore). Select your ROI across parts of the images, that are visible in both volumes.
   You can remove slices of your image stack with “Image” -> “Stacks” -> “Tools” -> “Slice Remover”. This will also reduce storage and computation time and possibly increase the accuracy.
4. Note down the new image dimensions.

*The following points can differ based on your selected image format. We choose the raw file and metaheader file combination.*

1. Save the image data: “File” -> “Save as” -> “Raw data…”
2. Create a *YourRawDataName.mhd* file inside the same folder where you saved the raw data.
   1. create a text file, copy and paste the lines below into the *.txt and adjust “DimSize” to the dimensions of your cropped images (order: x, y, z).
   2. adjust the “ElementDataFile” to your raw file name.
   3. check the “ElementType” to be the same as your raw data (8-bit unsigned integer: MET_UCHAR).
   4. save the *.txt and rename the file ending from “.txt” to “.mhd”.
   5. do this for both the reference and deformed state image.

ObjectType = Image

NDims = 3

BinaryData = True

BinaryDataByteOrderMSB = False

TransformMatrix = 1 0 0 0 1 0 0 0 1

Offset = 0 0 0

CenterOfRotation = 0 0 0

DimSize = 602 590 1024

ElementType = MET_USHORT

ElementDataFile = YourRawDataFile.raw

1. Check if you can open the raw data by dragging the *.mhd file into the FIJI menu bar.
   If the images don’t look as expected (e.g. stripes or noisy regular pattern), check the “DimSize” and “ElementType”.
2. Close FIJI

Guideline: calculating DVC using elastix

1. First download elastix from “elastix.dev”. You can add the elastix folder to the Windows PATH Environment Variables to enable global access.
2. Open a terminal or powershell at the position of your working folder for the DVC run (Windows: ALT+SHIFT+RIGHTCLICK -> “Open Terminal/Powershell here” or: Open Terminal and go to relevant folder by executing “cd *WORKINGFOLDERLOCATION*”).
3. Copy the relevant Parameter files into the working folder (e.g., parameters_Affine.txt & parameters_BSpline.txt). Example parameter files are e.g. provided at the end of this document.
   1. adjust the parameters inside the files to your needs (detailed information: see elastix manual). For the B-Spline registration parameters you should at least adjust the following:
      1. Metric
      2. FinalGridSpacingInVoxels
      3. NumberOfResolutions
      4. MaximumNumberOfIterations
      5. NumberOfSpatialSamples
      6. BSplineInterpolationOrder
      7. FinalBSplineInterpolationOrder
      8. ResultImageFormat
4. Run elastix using the command prompt below. **IMPORTANT: The file you later want the displacement field to be based on needs to be loaded as the fixed image.**You can run elastix with multiple parameter files. **If you e.g. want to do a non-affine B-Spline Registration, it is recommended to additionally run a affine registration before.** Elastix will then first do a affine registration and then perform the B-Spline registration based on the pre-aligned result images of the affine registration. An example command prompt could look like this:

elastix -f referenceImageFile.mhd -m deformedImageFile.mhd -p parameters_Affine.txt -p parameters_BSpline.txt -out OUTFOLDERLOCATION*(or just “.” to write in same folder where elastix is called)*

1. Run transformix using the command prompt in the same folder as you ran elastix (adjust file location if you wrote in a subfolder):
   transformix -def all -tp .\TransformParameters.1.txt -out .

Adjust the name of the transform parameter file (e.g. *.0.txt if elastix ran with only one parameter file).

Checking results

1. Quickly check your results using e.g. the VV viewer
   1. open VV
   2. “File” -> “Open image(s)” -> open your reference image
   3. “Overlay” -> “Add Overlay image to current image” -> select result0 of the elastix run

*You can now see the results of the affine registration.*

- 1. “File” -> “Open images” -> open another reference image
  2. “Overlay” -> “Add Overlay image to current image” -> select result1

*You can now see the results of the BSpline registration.*

- 1. “Overlay” -> “Add deformation field to current image” -> select the deformationfield.mhd that resulted from the transformix run
  2. go to “overlay” menu and increase th subsampling (e.g. to 20)

*You can now check if both registrations and the displacement field are plausible.*

Parameter files

Affine registration

// Example parameter file for rotation registration

// C-style comments: //

// The internal pixel type, used for internal computations

// Leave to float in general.

// NB: this is not the type of the input images! The pixel

// type of the input images is automatically read from the

// images themselves.

// This setting can be changed to "short" to save some memory

// in case of very large 3D images.

(FixedInternalImagePixelType "float")

(MovingInternalImagePixelType "float")

// Specify whether you want to take into account the so-called

// direction cosines of the images. Recommended: true.

// In some cases, the direction cosines of the image are corrupt,

// due to image format conversions for example. In that case, you

// may want to set this option to "false".

(UseDirectionCosines "true")

// **************** Main Components **************************

// The following components should usually be left as they are:

(Registration "MultiResolutionRegistration")

(Interpolator "BSplineInterpolator")

(ResampleInterpolator "FinalBSplineInterpolator")

(Resampler "DefaultResampler")

// These may be changed to Fixed/MovingSmoothingImagePyramid.

// See the manual.

(FixedImagePyramid "FixedSmoothingImagePyramid")

(MovingImagePyramid "MovingSmoothingImagePyramid")

// The following components are most important:

// The optimizer AdaptiveStochasticGradientDescent (ASGD) works

// quite ok in general. The Transform and Metric are important

// and need to be chosen careful for each application. See manual.

(Optimizer "AdaptiveStochasticGradientDescent")

(Transform "AffineTransform")

//(Metric "AdvancedMattesMutualInformation")

//(Metric "AdvancedMeanSquares")

(Metric "AdvancedNormalizedCorrelation")

// ***************** Transformation **************************

// Scales the rotations compared to the translations, to make

// sure they are in the same range. In general, it's best to

// use automatic scales estimation:

(AutomaticScalesEstimation "true")

// Automatically guess an initial translation by aligning the

// geometric centers of the fixed and moving.

(AutomaticTransformInitialization "true")

// Whether transforms are combined by composition or by addition.

// In generally, Compose is the best option in most cases.

// It does not influence the results very much.

(HowToCombineTransforms "Compose")

// ******************* Similarity measure *********************

// Number of grey level bins in each resolution level,

// for the mutual information. 16 or 32 usually works fine.

// You could also employ a hierarchical strategy:

//(NumberOfHistogramBins 16 32 64)

(NumberOfHistogramBins 32)

// If you use a mask, this option is important.

// If the mask serves as region of interest, set it to false.

// If the mask indicates which pixels are valid, then set it to true.

// If you do not use a mask, the option doesn't matter.

(ErodeMask "false")

// ******************** Multiresolution **********************

// The number of resolutions. 1 Is only enough if the expected

// deformations are small. 3 or 4 mostly works fine. For large

// images and large deformations, 5 or 6 may even be useful.

(NumberOfResolutions 3)

// The downsampling/blurring factors for the image pyramids.

// By default, the images are downsampled by a factor of 2

// compared to the next resolution.

// So, in 2D, with 4 resolutions, the following schedule is used:

//(ImagePyramidSchedule 8 8 4 4 2 2 1 1 )

// And in 3D:

//(ImagePyramidSchedule 8 8 8 4 4 4 2 2 2 1 1 1 )

// You can specify any schedule, for example:

//(ImagePyramidSchedule 4 4 4 3 2 1 1 1 )

// Make sure that the number of elements equals the number

// of resolutions times the image dimension.

// ******************* Optimizer ****************************

// Maximum number of iterations in each resolution level:

// 200-500 works usually fine for rigid registration.

// For more robustness, you may increase this to 1000-2000.

(MaximumNumberOfIterations 500)

// The step size of the optimizer, in mm. By default the voxel size is used.

// which usually works well. In case of unusual high-resolution images

// (eg histology) it is necessary to increase this value a bit, to the size

// of the "smallest visible structure" in the image:

//(MaximumStepLength 1.0)

// **************** Image sampling **********************

// Number of spatial samples used to compute the mutual

// information (and its derivative) in each iteration.

// With an AdaptiveStochasticGradientDescent optimizer,

// in combination with the two options below, around 2000

// samples may already suffice.

(NumberOfSpatialSamples 100000)

// Refresh these spatial samples in every iteration, and select

// them randomly. See the manual for information on other sampling

// strategies.

(NewSamplesEveryIteration "true")

(ImageSampler "Random")

// ************* Interpolation and Resampling ****************

// Order of B-Spline interpolation used during registration/optimisation.

// It may improve accuracy if you set this to 3. Never use 0.

// An order of 1 gives linear interpolation. This is in most

// applications a good choice.

(BSplineInterpolationOrder 3)

// Order of B-Spline interpolation used for applying the final

// deformation.

// 3 gives good accuracy; recommended in most cases.

// 1 gives worse accuracy (linear interpolation)

// 0 gives worst accuracy, but is appropriate for binary images

// (masks, segmentations); equivalent to nearest neighbor interpolation.

(FinalBSplineInterpolationOrder 3)

//Default pixel value for pixels that come from outside the picture:

(DefaultPixelValue 0)

// Choose whether to generate the deformed moving image.

// You can save some time by setting this to false, if you are

// only interested in the final (nonrigidly) deformed moving image

// for example.

(WriteResultImage "true")

// The pixel type and format of the resulting deformed moving image

(ResultImagePixelType "unsigned short")

(ResultImageFormat "mhd")

B-Spline registration

// Example parameter file for B-spline registration

// C-style comments: //

// The internal pixel type, used for internal computations

// Leave to float in general.

// NB: this is not the type of the input images! The pixel

// type of the input images is automatically read from the

// images themselves.

// This setting can be changed to "short" to save some memory

// in case of very large 3D images.

(FixedInternalImagePixelType "float")

(MovingInternalImagePixelType "float")

// The dimensions of the fixed and moving image

// Up to elastix 4.5 this had to be specified by the user.

// From elastix 4.6, this is not necessary anymore.

//(FixedImageDimension 2)

//(MovingImageDimension 2)

// Specify whether you want to take into account the so-called

// direction cosines of the images. Recommended: true.

// In some cases, the direction cosines of the image are corrupt,

// due to image format conversions for example. In that case, you

// may want to set this option to "false".

(UseDirectionCosines "true")

// **************** Main Components **************************

// The following components should usually be left as they are:

(Registration "MultiResolutionRegistration")

(Interpolator "BSplineInterpolator")

(ResampleInterpolator "FinalBSplineInterpolator")

(Resampler "DefaultResampler")

// These may be changed to Fixed/MovingSmoothingImagePyramid.

// See the manual.

(FixedImagePyramid "FixedSmoothingImagePyramid")

(MovingImagePyramid "MovingSmoothingImagePyramid")

// The following components are most important:

// The optimizer AdaptiveStochasticGradientDescent (ASGD) works

// quite ok in general. The Transform and Metric are important

// and need to be chosen careful for each application. See manual.

(Optimizer "AdaptiveStochasticGradientDescent")

(Transform "BSplineTransform")

//(Metric "AdvancedMattesMutualInformation")

(Metric "AdvancedNormalizedCorrelation")

//(Metric "AdvancedMeanSquares")

// ***************** Transformation **************************

// The control point spacing of the bspline transformation in

// the finest resolution level. Can be specified for each

// dimension differently. Unit: mm.

// The lower this value, the more flexible the deformation.

// Low values may improve the accuracy, but may also cause

// unrealistic deformations. This is a very important setting!

// We recommend tuning it for every specific application. It is

// difficult to come up with a good 'default' value.

//(FinalGridSpacingInPhysicalUnits 8)

// Alternatively, the grid spacing can be specified in voxel units.

// To do that, uncomment the following line and comment/remove

// the FinalGridSpacingInPhysicalUnits definition.

(FinalGridSpacingInVoxels 25)

// By default the grid spacing is halved after every resolution,

// such that the final grid spacing is obtained in the last

// resolution level. You can also specify your own schedule,

// if you uncomment the following line:

//(GridSpacingSchedule 2.0 1.0)

// This setting can also be supplied per dimension.

// Whether transforms are combined by composition or by addition.

// In generally, Compose is the best option in most cases.

// It does not influence the results very much.

(HowToCombineTransforms "Compose")

// ******************* Similarity measure *********************

// Number of grey level bins in each resolution level,

// for the mutual information. 16 or 32 usually works fine.

// You could also employ a hierarchical strategy:

//(NumberOfHistogramBins 16 32 64)

(NumberOfFixedHistogramBins 32)

// If you use a mask, this option is important.

// If the mask serves as region of interest, set it to false.

// If the mask indicates which pixels are valid, then set it to true.

// If you do not use a mask, the option doesn't matter.

(ErodeMask "false")

// ******************** Multiresolution **********************

// The number of resolutions. 1 Is only enough if the expected

// deformations are small. 3 or 4 mostly works fine. For large

// images and large deformations, 5 or 6 may even be useful.

(NumberOfResolutions 5)

// The downsampling/blurring factors for the image pyramids.

// By default, the images are downsampled by a factor of 2

// compared to the next resolution.

// So, in 2D, with 4 resolutions, the following schedule is used:

//(ImagePyramidSchedule 8 8 4 4 2 2 1 1 )

// And in 3D:

//(ImagePyramidSchedule 8 8 8 4 4 4 2 2 2 1 1 1 )

// You can specify any schedule, for example:

//(ImagePyramidSchedule 4 4 4 3 2 1 1 1 )

// Make sure that the number of elements equals the number

// of resolutions times the image dimension.

// ******************* Optimizer ****************************

// Maximum number of iterations in each resolution level:

// 200-2000 works usually fine for nonrigid registration.

// The more, the better, but the longer computation time.

// This is an important parameter!

(MaximumNumberOfIterations 1000)

// The step size of the optimizer, in mm. By default the voxel size is used.

// which usually works well. In case of unusual high-resolution images

// (eg histology) it is necessary to increase this value a bit, to the size

// of the "smallest visible structure" in the image:

//(MaximumStepLength 1.0)

// **************** Image sampling **********************

// Number of spatial samples used to compute the mutual

// information (and its derivative) in each iteration.

// With an AdaptiveStochasticGradientDescent optimizer,

// in combination with the two options below, around 2000

// samples may already suffice.

(NumberOfSpatialSamples 100000)

// Refresh these spatial samples in every iteration, and select

// them randomly. See the manual for information on other sampling

// strategies.

(NewSamplesEveryIteration "true")

(ImageSampler "Random")

// ************* Interpolation and Resampling ****************

// Order of B-Spline interpolation used during registration/optimisation.

// It may improve accuracy if you set this to 3. Never use 0.

// An order of 1 gives linear interpolation. This is in most

// applications a good choice.

(BSplineInterpolationOrder 3)

// Order of B-Spline interpolation used for applying the final

// deformation.

// 3 gives good accuracy; recommended in most cases.

// 1 gives worse accuracy (linear interpolation)

// 0 gives worst accuracy, but is appropriate for binary images

// (masks, segmentations); equivalent to nearest neighbor interpolation.

(FinalBSplineInterpolationOrder 3)

//Default pixel value for pixels that come from outside the picture:

(DefaultPixelValue 0)

// Choose whether to generate the deformed moving image.

// You can save some time by setting this to false, if you are

// not interested in the final deformed moving image, but only

// want to analyze the deformation field for example.

(WriteResultImage "true")

// The pixel type and format of the resulting deformed moving image

(ResultImagePixelType "unsigned short")

(ResultImageFormat "mhd")

Guideline: Pre-processing with Avizo™


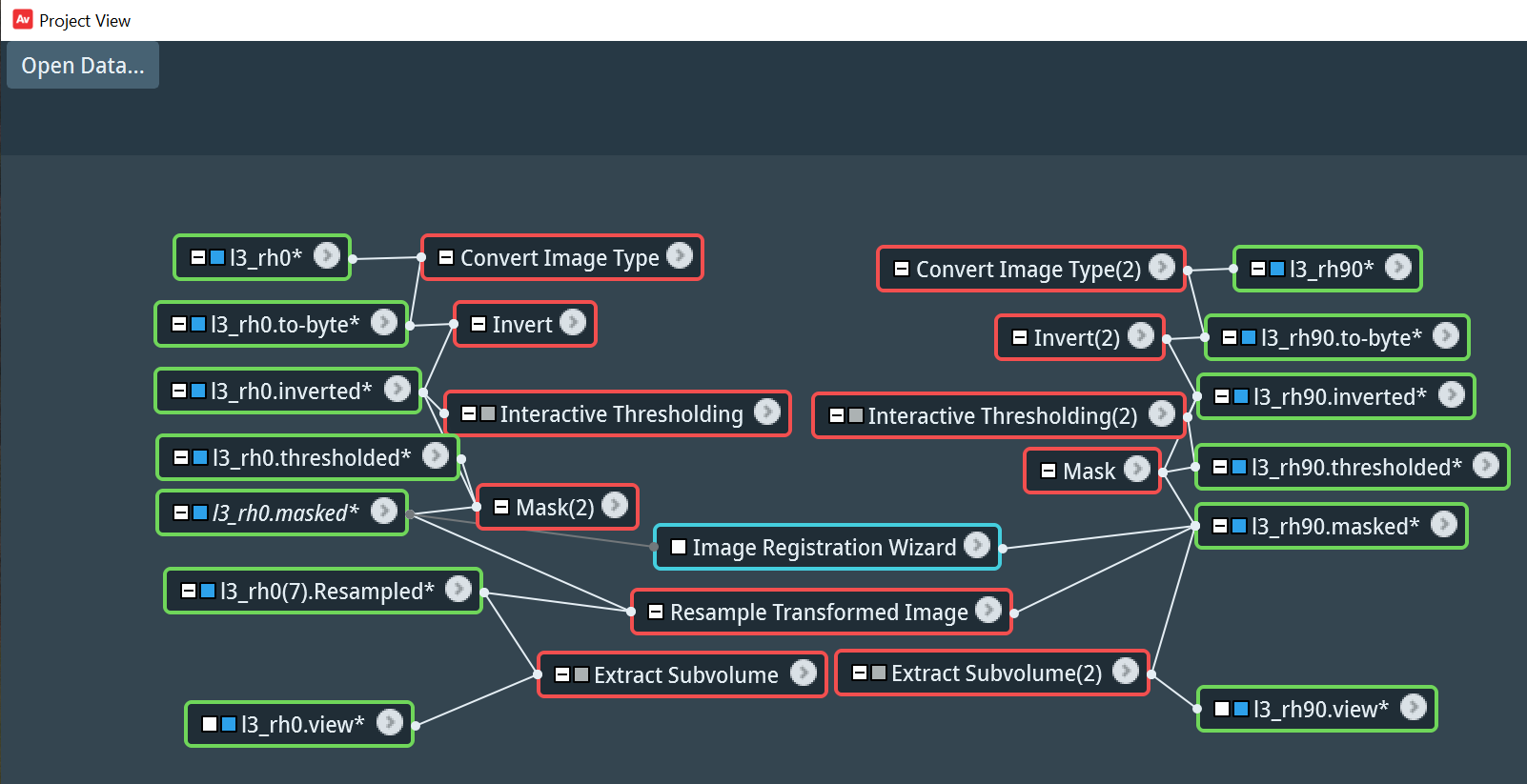


**Figure S12:** Project view after pre-processing using Avizo™.

1. Load the datasets (here: l3 at approx. 0 % and 90 % relative humidity).
2. Convert the image type to 8 bit.
3. Depending on the reconstruction, the data has to be inverted.
4. Apply an “Interactive Thresholding” on both volumes.
5. “Mask” both volumes using the volume and the threshold.


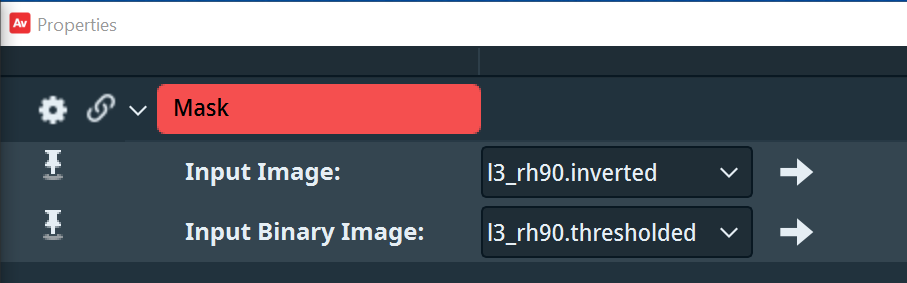


**Figure S13:** Applying a mask in Avizo™.

1. Use the “Image registration Wizard” on the masked datasets to rigidly align them.
   1. Open the image registration wizard using the reference image, as you cannot change the reference in the properties window.
   2. One volume name will be written in italics 🡪 that’s the realigned volume.
2. Run “Resample transformed image” to resample the realigned volume.
   1. Open the arrow beside the Data and choose the other status (should be the reference image) as a reference.


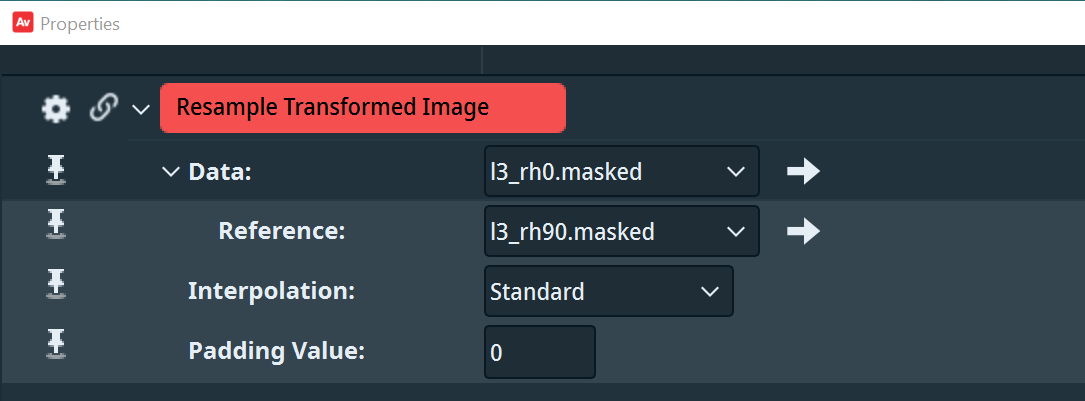


**Figure S14**: Resampling of the sample to change the room coordinates.

- 1. Now the voxel positions of both datasets are identical to each other.

1. “Extract Subvolume” of both volumes to crop most of the ambient air.
   1. Use the global coordinates for both datasets!
      1. Here, as the voxel positions are identical, you can use the local coordinates.
2. With this, the files “l3_rh0.view” and “l3_rh90.view” are ready to be analysed.

Guideline: DVC using Avizo™

Step 1: Importing datasets

1. Load the datasets.

Step 2: Local DVC


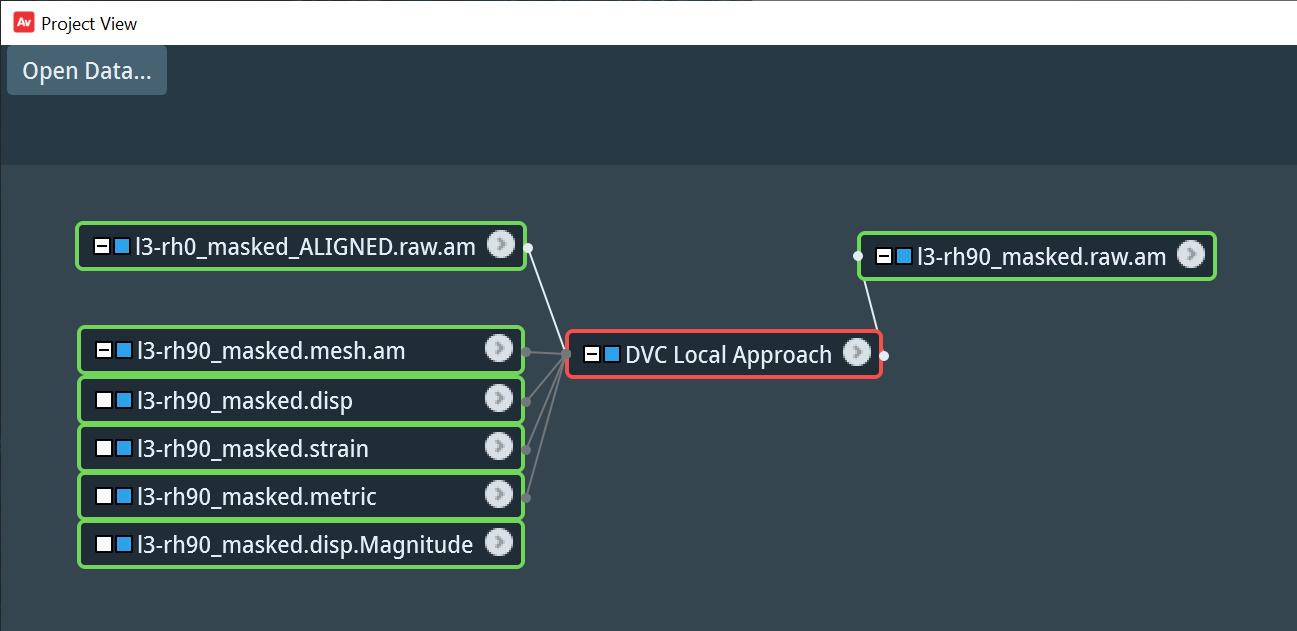


**Figure S15:** Project View after calculating the DVC Local Approach.

1. Run “DVC local Approach”.
   1. Select the reference and the deformed volume.
   2. Sub-volume size: approx. 10 μm (depends on the voxel size of the sample -> Here: approx. 100 nm, thus, length 100) 🡪 a coarse grid is sufficient.
   3. Metric: Correlation (threshold: 0.7).
   4. Transform: Translation and Rotation.


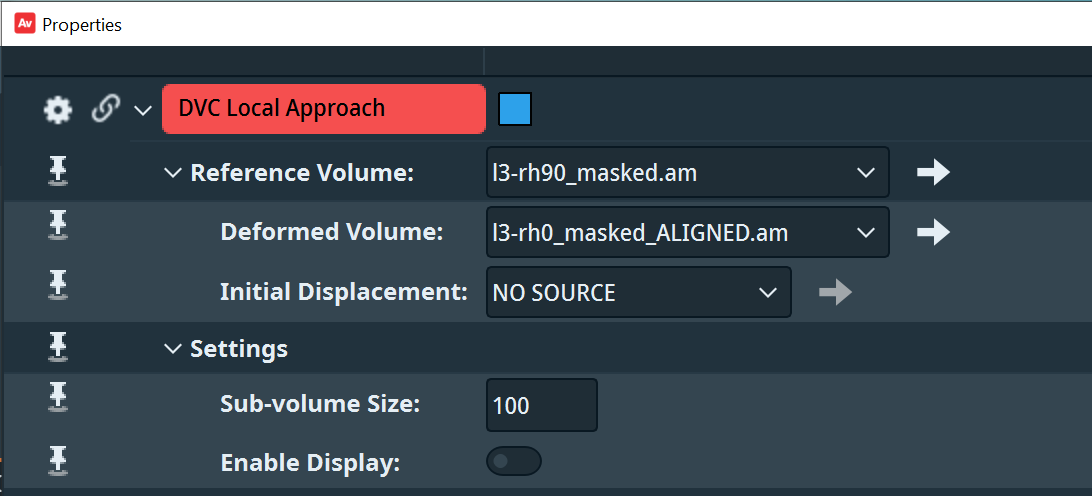


**Figure S16:** Settings of the DVC Local Approach in Avizo™.

1. The results of the “DVC Local Approach” can be visualised using “Displacement View” on the X.disp or the X.strain file.
   1. For better visualisation, the colormap can be changed to “physics.icol” in the X.disp.magnitude file.


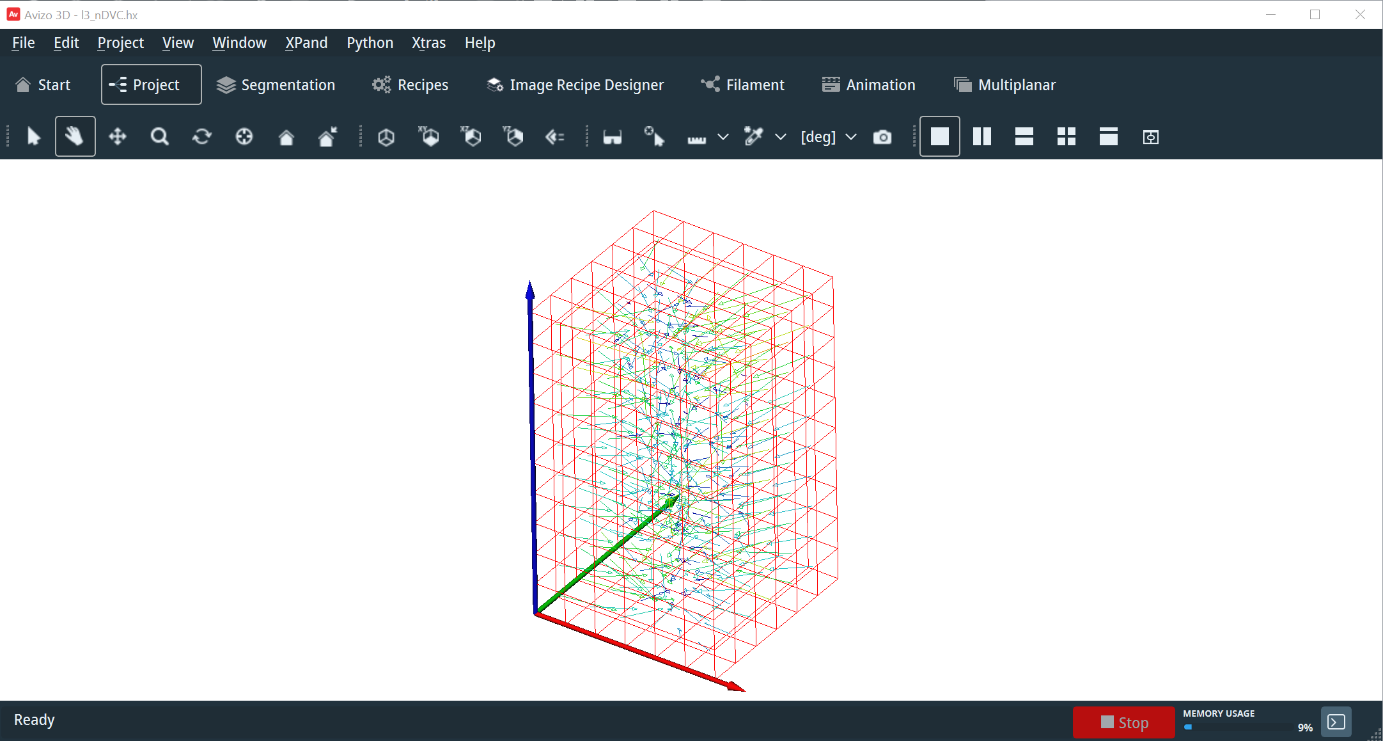


**Figure S17:** Displacement view of the resulting displacement field of the DVC Local Approach with Axes and the reference grid.

Step 3: Creating the mesh for the global DVC


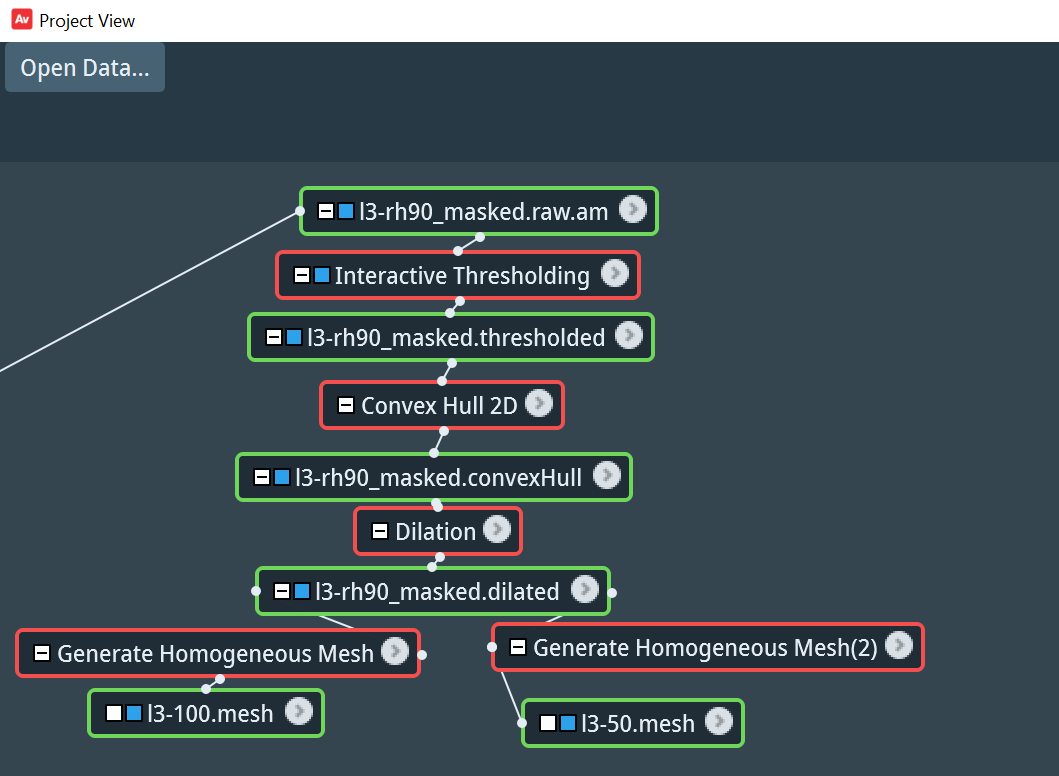


**Figure S18:** Project View after creating the two meshes for the DVC Global Approach.

1. Apply an “Interactive Thresholding” on the reference image.
2. Create an “Convex Hull 2D” using the XY-planes.
3. Dilate the convex hull by 8; Settings: Type: Cube, Interpretation: 3D, Neighbourhood: 26.
4. “Create homogenous mesh” with length approx. 10 μm (depends on the voxel size of the sample -> Here: approx. 100nm, thus, length 100).
   1. A coarse mesh is created.
   2. Fix the mesh using the console with: “name of the mesh“ cleanUp.
5. “Create homogenous mesh” with length approx. 5 μm (length 50).
   1. A fine mesh is created.
   2. Fix the mesh using the console: “name of the mesh“ cleanUp.
6. Control the mesh.
   1. “Tetra Grid View” on the mesh and “Volume Rendering” on the reference volume.
   2. The sample should be covered in the mesh.


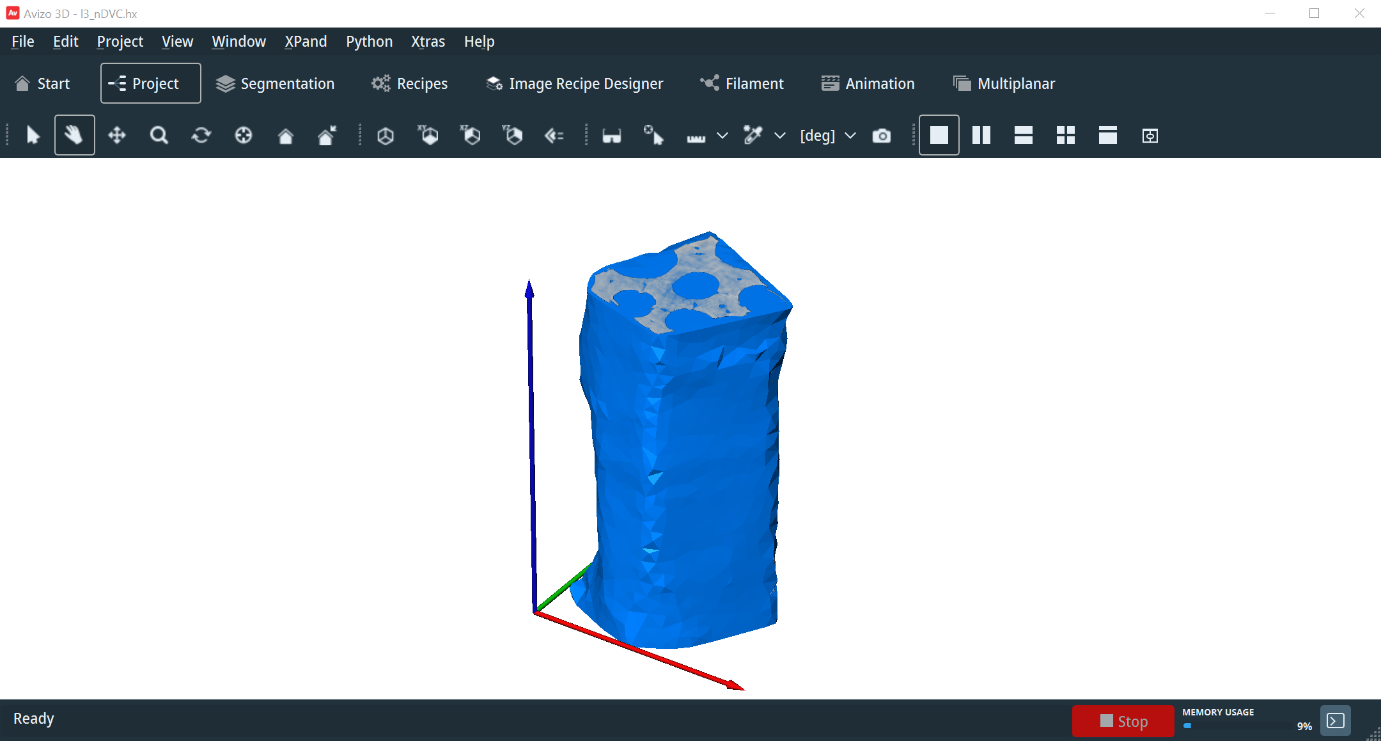


**Figure S19:** Volume Rendering of the reference volume and Tetra Grid View of the fine mesh.

Step 4: Global DVC


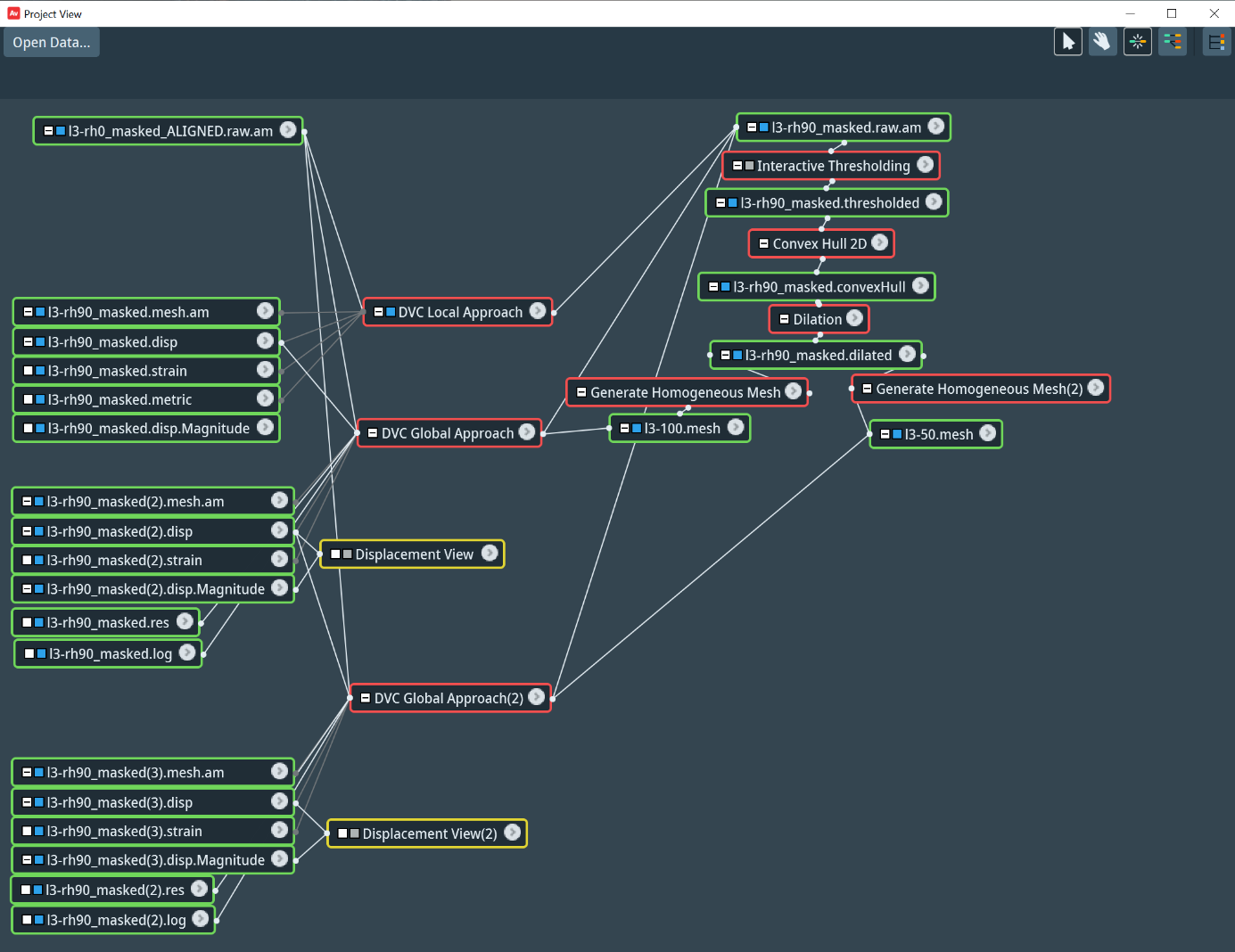


**Figure S10:** Project View after running 2 DVC Global Approaches.

1. Run “DVC Global Approach” (1).
   1. Select the reference and the deformed volume.
   2. Reference mesh: coarse mesh.
   3. Initial Displacement: .disp result of the local DVC.
   4. Settings:
      1. Max Iterations: 200 (or 500 when a better fit is intended 🡪 instead a second global DVC with the same volumes and the displ. Of the first global DVC can be run).
      2. Convergence criterion: 0.001
   5. Regularization
      1. Has to be active. The standard value is sufficient most of the times. Can be increased for a fine mesh with large deformations.


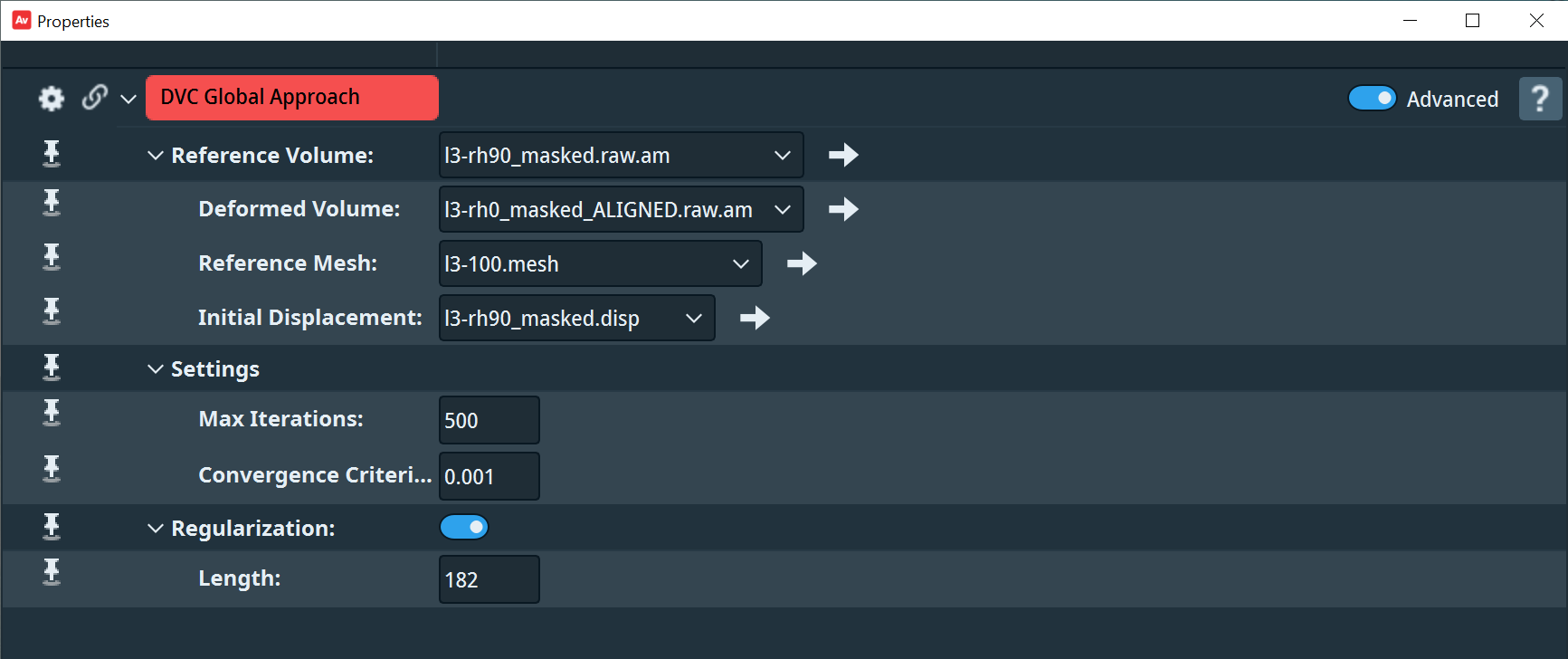


**Figure S11:** Settings for the first DVC Global Approach.

- 1. While running: check the displacement increment in the console (or: check the displacement increment after the DVC is finished).
     1. Displacement increment corresponds to the fit of the deformation found.
     2. Displacement of 0.001 as the goal.
  2. If the displacement increment is not converging towards 0, the DVC can be considered failed and the mesh has to be looked upon. See “Error Correction” in this guideline.


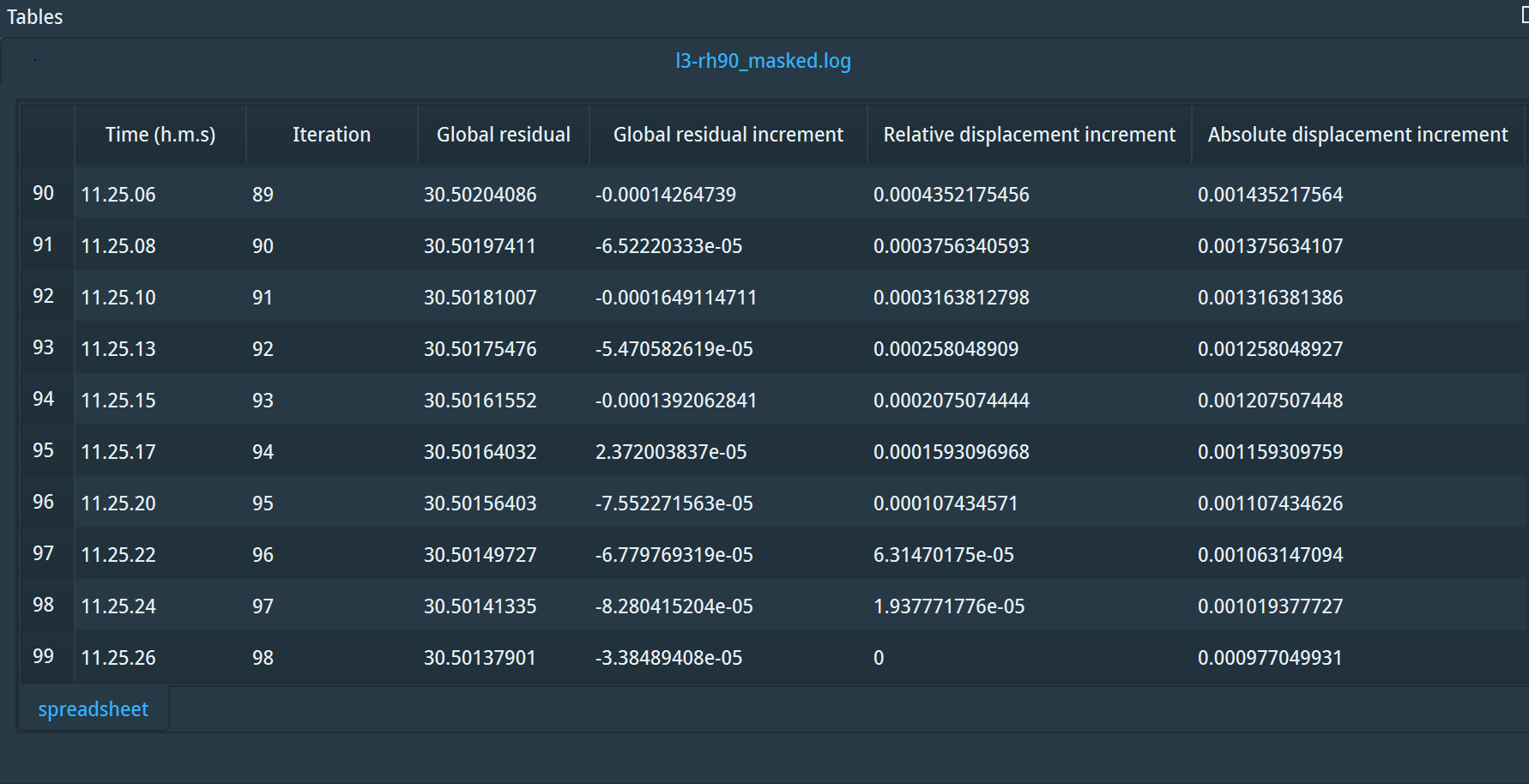


**Figure S12:** Spreadsheet containing the displacement increments for each iteration during the DVC Global Approach.

1. Run “Global DVC Approach” (2).
   1. Select the reference and the deformed volume.
   2. Reference mesh: Fine mesh.
   3. Initial Displacement: .disp result of the coarse Global DVC.
   4. Settings:
      1. Max Iterations: 200 (or 500 when a better fit is intended 🡪 instead a second Global DVC with the same volumes and the displ. Of the first global dvc can be run).
      2. Convergence criterion: 0.001
   5. Regularization
      1. Has to be active. The standard value is sufficient most of the times. Can be increased for a fine mesh with large deformations.
   6. While running: check the displacement increment in the console (or: check the displacement increment after the DVC is finished).
      1. Displacement increment corresponds to the fit of the deformation found.
      2. Displacement of 0.001 as the goal.
   7. If the displacement increment is not converging towards 0, the DVC can be considered failed and the mesh has to be looked upon. See “Error Correction” in this guideline.

Step 5: Understanding the results of the DVC


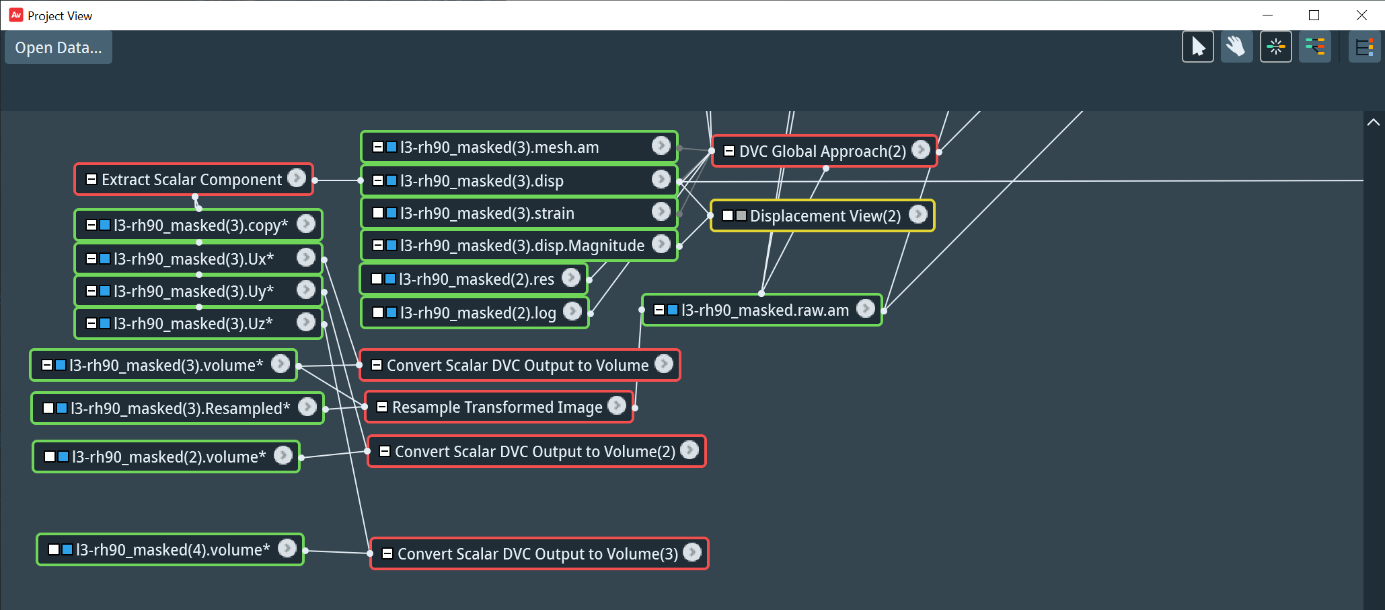


**Figure S13:** Project view after extracting the displacement along x in the original voxel resolution.

.disp file

1. The .disp file contains the 3D displacement field.
2. Vectors can be depicted using “Displacement View”.


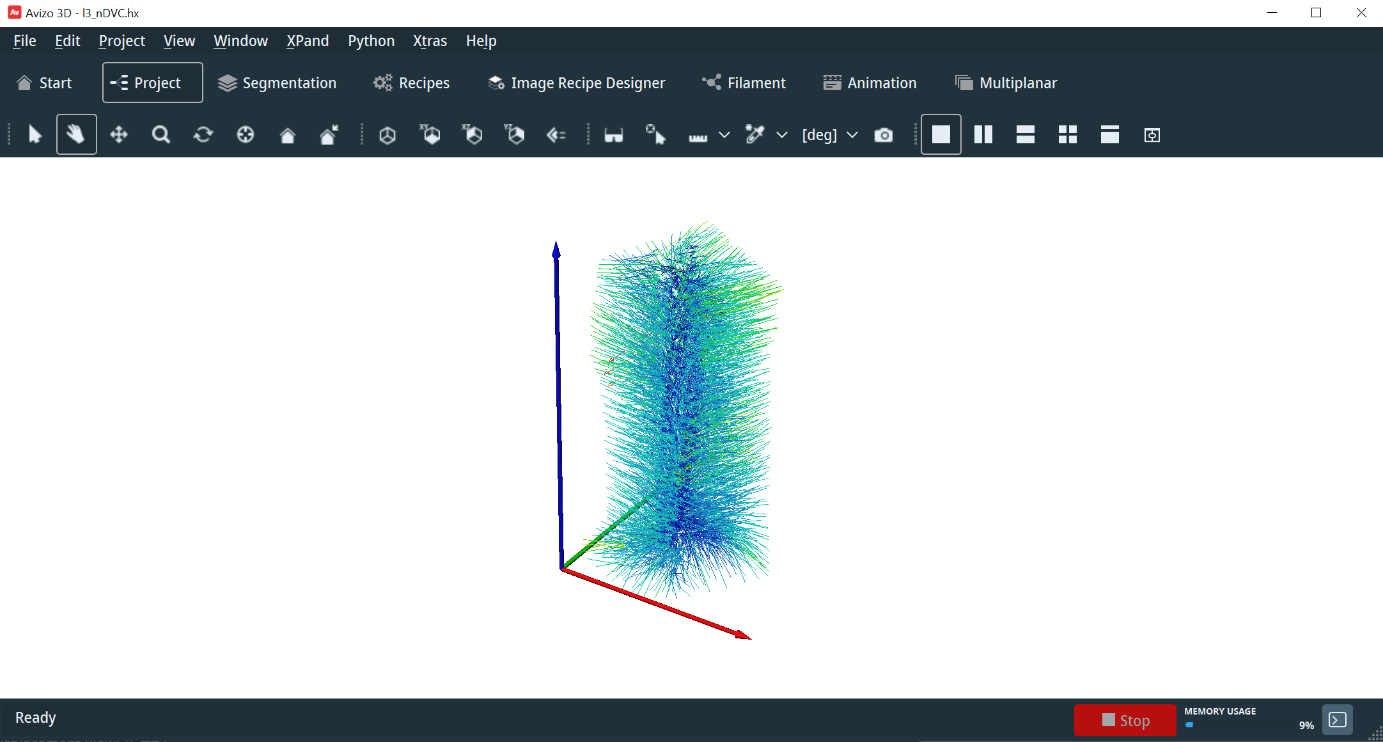


**Figure S14:** Visualisation of the displacement field as vectors.

1. Spreadsheet can be extracted by “Convert DVC Output to Spreadsheet (Convert)”.
   1. Export spreadsheet, to create a .csv file, otherwise, it is only going to be saved as an .am file.
2. Displacement according the axes can be extracted by “Extract Scalar Component”.
3. “Convert Scalar Component to Volume” with volume dimensions close to the initial dataset.
   1. Accounts for the resolution of the new voxel-based image, the actual image size stays the same.
4. “Resample transformed image” to interpolate the new voxel-based image to the voxels of the initial image.
   1. Use the initial reference dataset as a reference by clicking on the small arrow next to the “data”.
   2. Choose “nan” for “Padding value” and “Standard” for “Interpolation”.


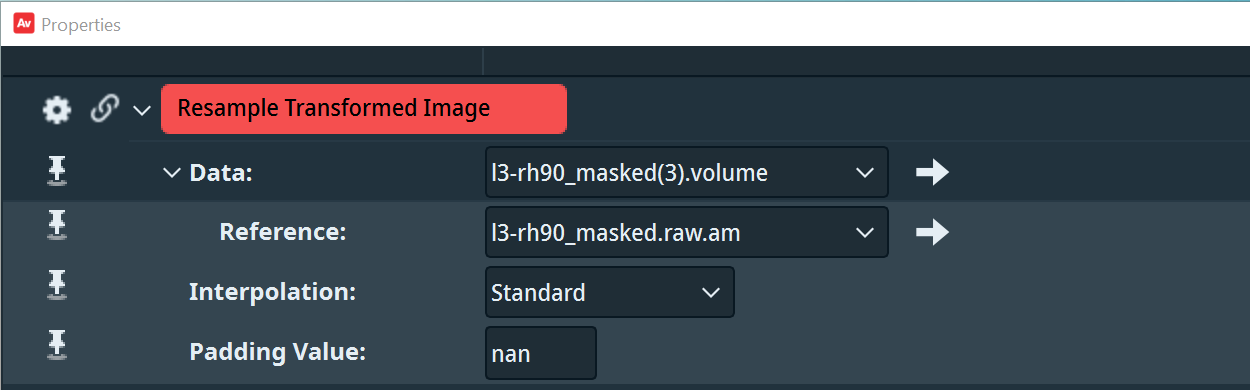


**Figure S15:** Resampling the displacement field along the x-axis to the initial voxel resolution.

1. “Mask” using the threshold image of the reference data.
2. Resulting displacement fields can be exported as various datatypes.

.strain file

1. Same as with the displacement field but depicting the strains.
2. When extracting the scalar component, Exx, Eyy, Ezz, Exy, Exz, Eyz and E1, E2, E3 can be chosen.
   1. Exx, Eyy, … are the green lagrangian strains along the axes.
   2. E1, E2 and E3 are the principal strains.


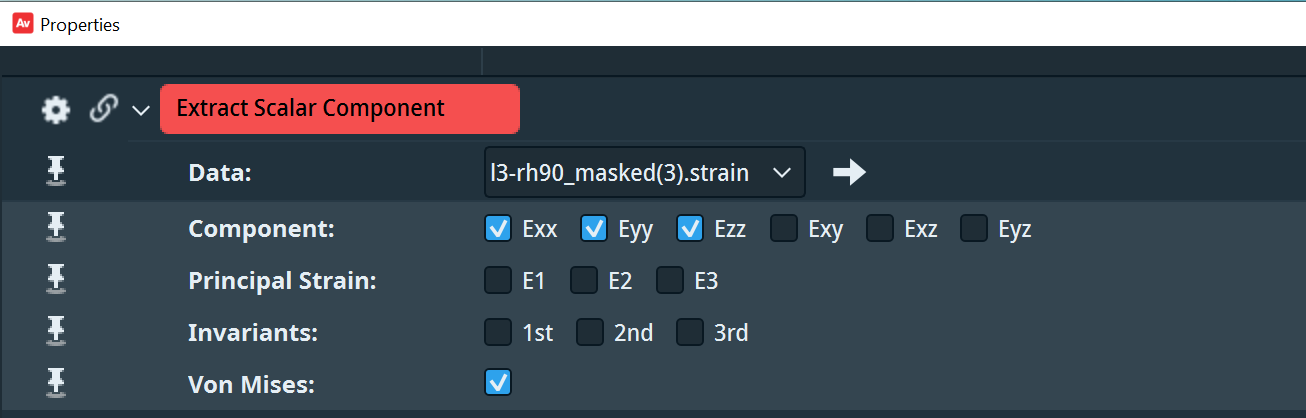


**Figure S16:** Settings upon extracting directed strains.

1. Beware: the masked version of the voxel-based dataset may contain “inf values”
   1. One way to get rid of the “inf values” is to export the file to manually delete the values using python.

.res file

1. The residuals file shows the accuracy between the calculated displacement and the real displacement by applying the displacement field onto the reference volume and subtracting the deformed volume.


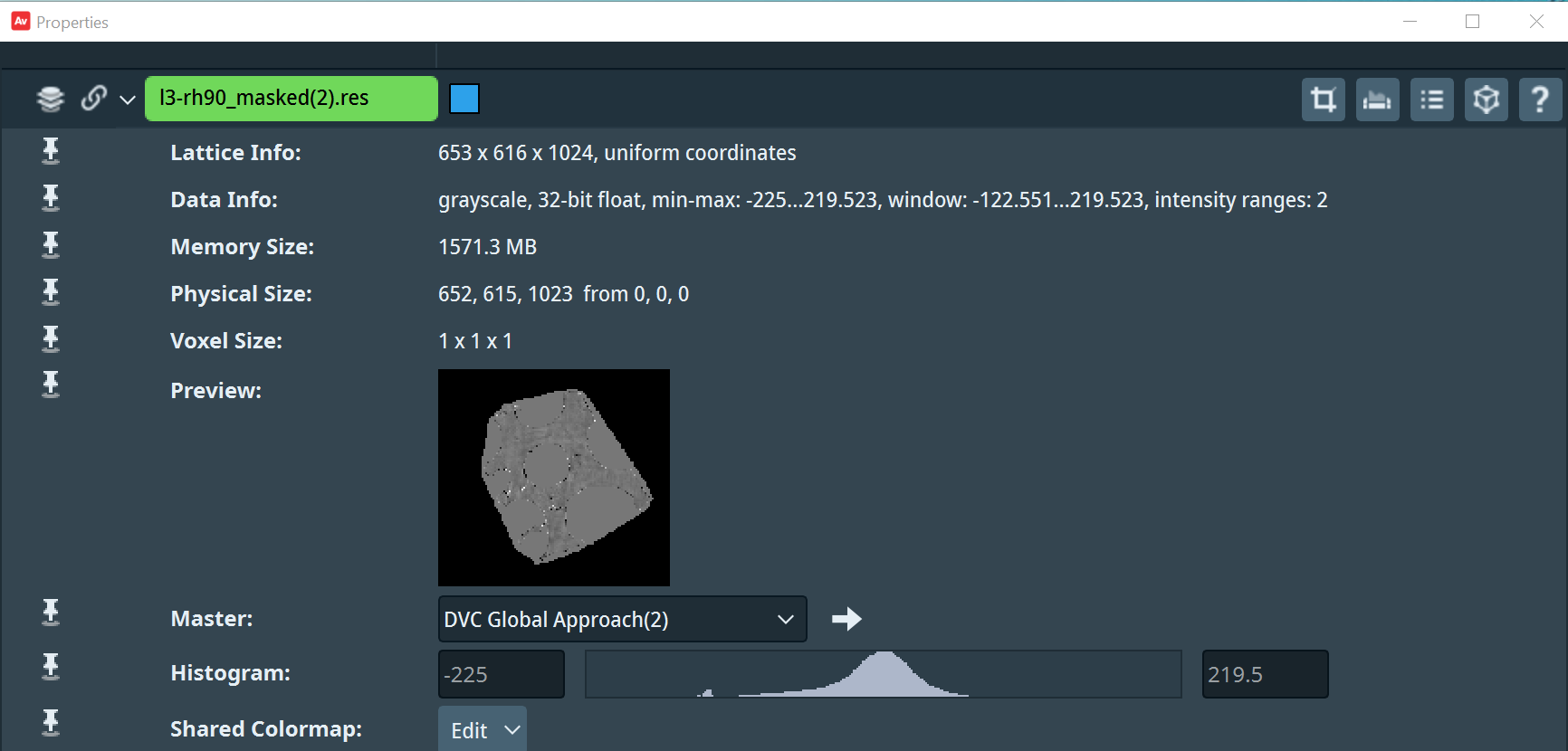


**Figure S17:** Properties window of the residuals file presenting the accuracy of the DVC.

1. The histogram shows the standard deviation.

Step 6: Visualisation of the results

There are three distinct options available for visualisation.


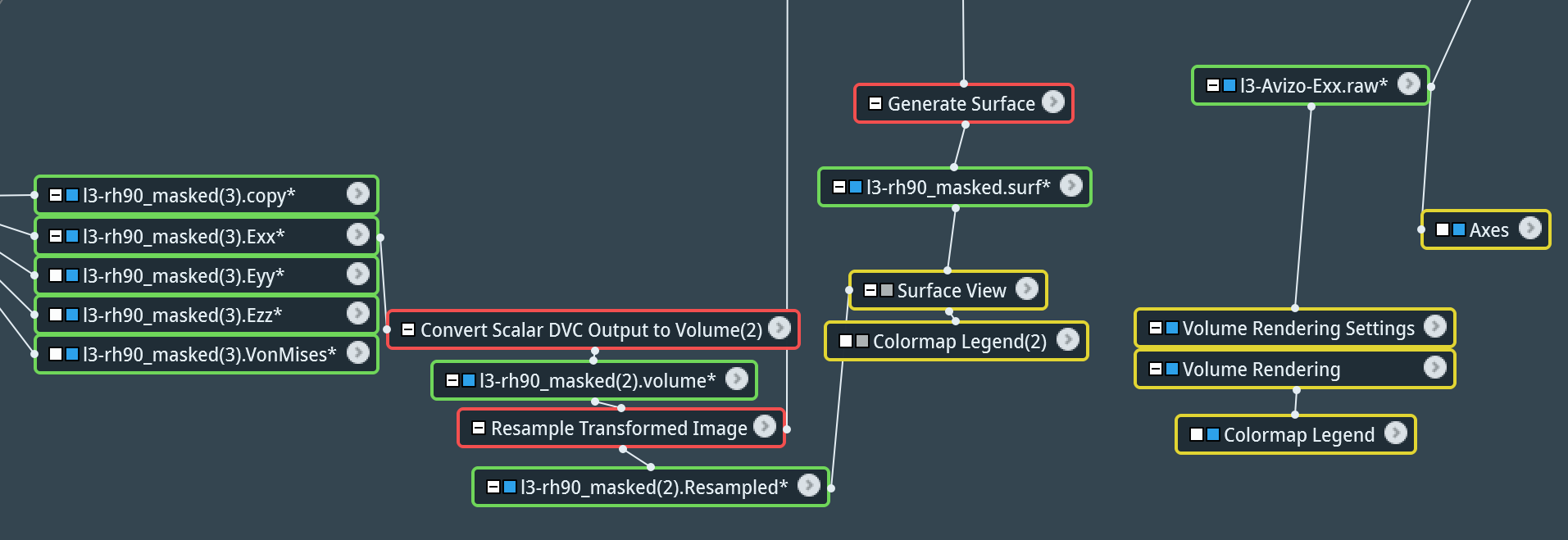


**Figure S18:** Project view containing the necessary steps for three different visualizations of the DVC results.

1. Visualising the convex hull of the displacement or the strain by applying a “Volume Rendering” to the displacement field or the strain field components.
   1. Use the resampled displacement or strain component (Step 5) as a reference.


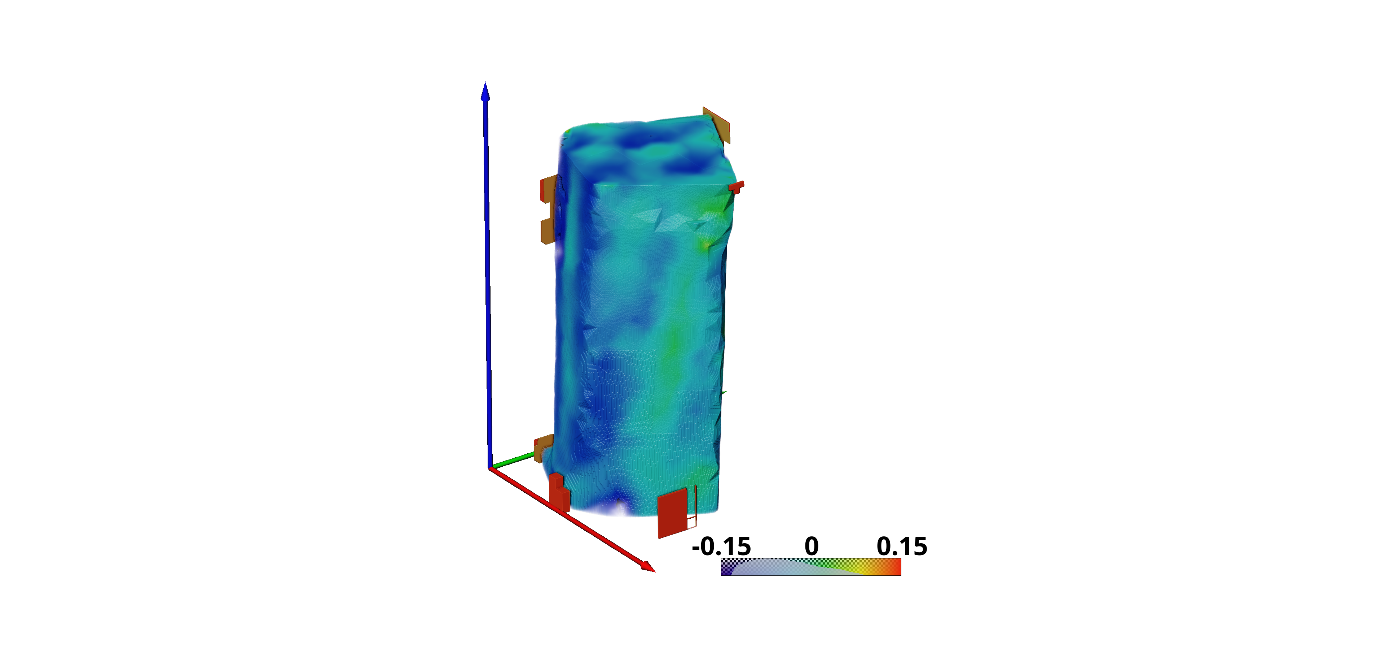


**Figure S19:** Volume rendering of the strain along the x-axis immediately after converting the strain field into a volume.

1. Visualising the colormap of the displacement or strain component onto the sample surface.
   1. Use “Generate Surface” on the threshold image of Step 3.1.
   2. Visualise the surface with “Surface View” and choose the resampled displacement or strain component as a colormap.


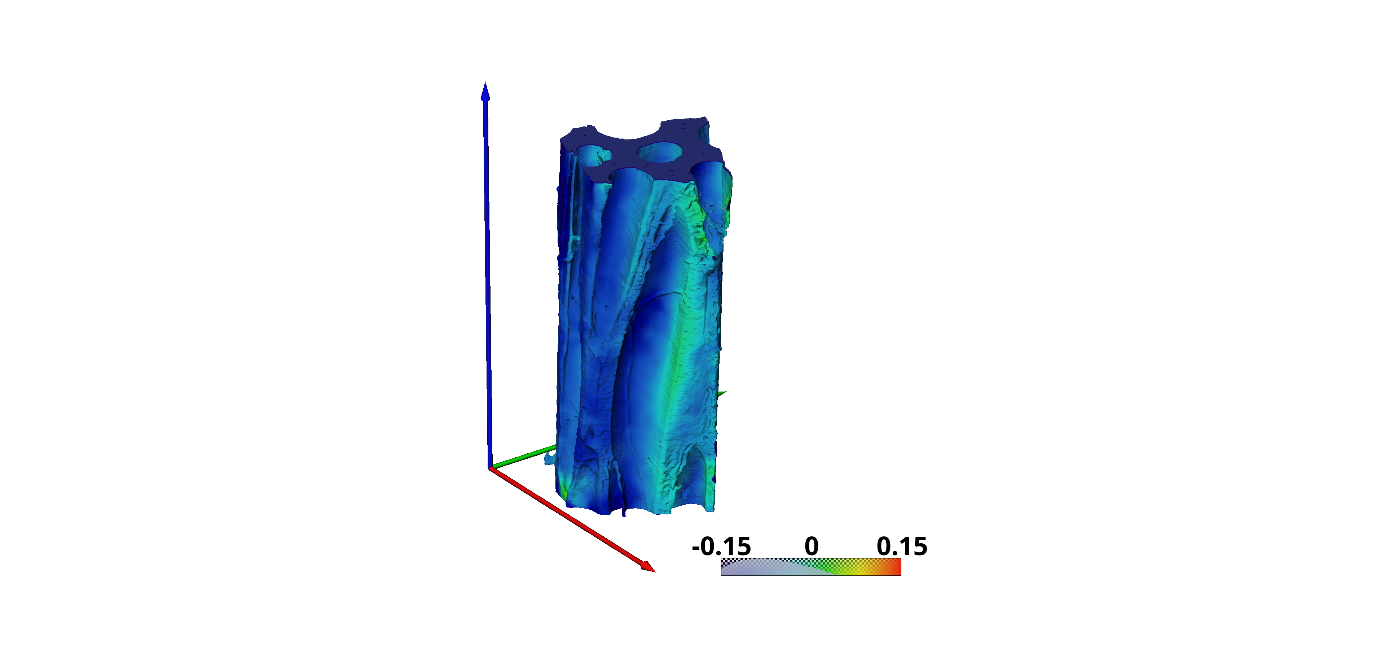


**Figure S20:** Mapping the strain field along the x-axis onto the sample surface (corlomap).

1. Visualise the masked volumes (Step 5.3) with “Volume Rendering”.


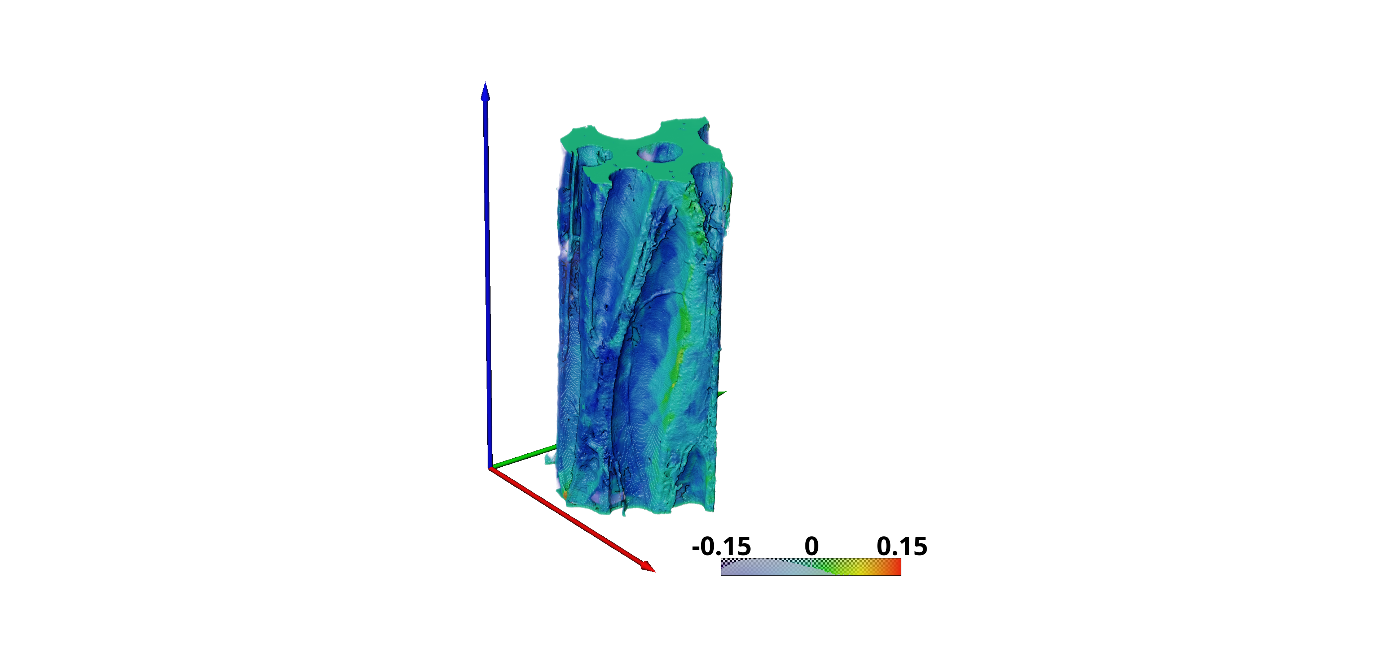


**Figure S21:** Volume rendering of the strain along the x-axis after masking the convex hull.
